# Effect of Plant Versus Animal Protein on Muscle Mass, Strength, Physical Performance, and Sarcopenia: A Systematic Review and Meta-analysis of Randomized Controlled Trials

**DOI:** 10.1093/nutrit/nuae200

**Published:** 2025-01-15

**Authors:** Rachel J Reid-McCann, Sarah F Brennan, Nicola A Ward, Danielle Logan, Michelle C McKinley, Claire T McEvoy

**Affiliations:** Nutrition and Metabolism Research Group, Centre for Public Health, Queen’s University Belfast Royal Victoria Hospital, Belfast BT12 6BJ, United Kingdom; Nutrition and Metabolism Research Group, Centre for Public Health, Queen’s University Belfast Royal Victoria Hospital, Belfast BT12 6BJ, United Kingdom; Nutrition and Metabolism Research Group, Centre for Public Health, Queen’s University Belfast Royal Victoria Hospital, Belfast BT12 6BJ, United Kingdom; Nutrition and Metabolism Research Group, Centre for Public Health, Queen’s University Belfast Royal Victoria Hospital, Belfast BT12 6BJ, United Kingdom; Nutrition and Metabolism Research Group, Centre for Public Health, Queen’s University Belfast Royal Victoria Hospital, Belfast BT12 6BJ, United Kingdom; Nutrition and Metabolism Research Group, Centre for Public Health, Queen’s University Belfast Royal Victoria Hospital, Belfast BT12 6BJ, United Kingdom

**Keywords:** dietary protein, sarcopenia, muscle mass, strength, physical performance, meta-analysis

## Abstract

**Context:**

Dietary protein is recommended for sarcopenia—a debilitating condition of age-related loss of muscle mass and strength that affects 27% of older adults. The effects of protein on muscle health may depend on protein quality.

**Objective:**

The aim was to synthesize randomized controlled trial (RCT) data comparing plant with animal protein for muscle health.

**Data Sources:**

Forty-three eligible RCTs were sourced from Medline, Embase, Scopus, Web of Science, and CENTRAL databases.

**Data Extraction:**

Four reviewers (R.J.R.-M., S.F.B., N.A.W., D.L.) extracted data from RCTs (study setting, population, intervention characteristics, outcomes, summary statistics) and conducted quality assessment using the Cochrane Risk of Bias 2.0.

**Data Analysis:**

Standardized mean differences (SMDs) (95% CIs) were combined using a random-effects meta-analysis and forest plots were generated. I^2^ statistics were calculated to test for statistical heterogeneity.

**Conclusion:**

Thirty RCTs (70%) were eligible for meta-analysis and all examined muscle mass outcomes. Compared with animal protein, plant protein resulted in lower muscle mass following the intervention (SMD = –0.20; 95% CI: –0.37, –0.03; P = .02), with stronger effects in younger (<60 years; SMD = –0.20; 95% CI: –0.37, –0.03; P = .02) than in older (≥60 years; SMD = –0.05; 95% CI: –0.32, 0.23; P = .74) adults. There was no pooled effect difference between soy and milk protein for muscle mass (SMD = –0.02; 95% CI: –0.20, 0.16; P = .80) (n = 17 RCTs), yet animal protein improved muscle mass compared with non-soy plant proteins (rice, chia, oat, and potato; SMD = –0.58; 95% CI: –1.06, –0.09; P = .02) (n = 5 RCTs) and plant-based diets (SMD = –0.51; 95% CI: –0.91, –0.11; P = .01) (n = 7 RCTs). No significant difference was found between plant or animal protein for muscle strength (n = 14 RCTs) or physical performance (n = 5 RCTs). No trials examined sarcopenia as an outcome. Animal protein may have a small beneficial effect over non-soy plant protein for muscle mass; however, research into a wider range of plant proteins and diets is needed.

**Systematic Review Registration:**

PROSPERO registration no. CRD42020188658.

## INTRODUCTION

Sarcopenia is a debilitating condition characterized by loss of muscle mass and strength and is estimated to affect up to 27% of older adults over the age of 60 years.^1^ Muscle mass is lost at a rate of 0.4% to 0.5% per year, increasing to 0.6% to 1% per year after the age of 75.^2^ Muscle strength is lost at an even faster rate, at 3% to 4% per year in men and 2.5% to 3% in women after the age of 75.^2^ Sarcopenia is associated with numerous adverse outcomes, including falls, frailty, depression, hospitalization, and death^3^; therefore, there is a critical need to identify effective interventions for the prevention or management of sarcopenia in an aging population.

Adults with low muscle mass and strength tend to consume less dietary protein than others with normal muscle status,^4–6^ indicating that dietary protein may be an important modifiable risk factor for sarcopenia. Adequate dietary protein (1.0 to 1.5 g/kg of body weight per day [g/kg bw/d]),^7^ either through a protein-rich diet or protein supplementation, alongside resistance training (RT) is recommended as a primary prevention strategy for sarcopenia.^8^^,^^9^ However, the role of protein source remains unclear. Protein from plant sources is generally considered to be of a lower quality, with a lower Digestible Indispensable Amino Acid Score (DIAAS) than animal protein comparators, on average.^10^ Essential amino acids (EAAs), especially branched-chain amino acids (BCAAs) such as leucine, are important in the regulation of muscle protein synthesis (MPS).^11^ A lower concentration of BCAAs such as leucine in plant proteins may result in a less potent effect on improvement in muscle mass in older adults who are most at risk of sarcopenia. Furthermore, there is evidence that a plant protein meal with a similar amino acid profile to an omnivorous meal fails to stimulate postprandial MPS rate while the omnivorous meal succeeds, suggesting that the structure, and thus digestibility of the protein, may be as important as amino acid content.^12^ It is important to understand how plant proteins compare with animal proteins for supporting muscle and functional health outcomes, especially considering the increased popularity of plant-based meat alternatives in the replacement of traditional animal proteins.

A small number of systematic reviews have aimed to investigate this research question previously; however, the syntheses did not include plant proteins other than soy,^13^^,^^14^ while there are indeed a growing number of trials that aim to investigate the effects of a more diverse range of plant proteins on anabolic stimulus and functional health.^12^^,^^15–20^ It is important to include these trials in systematic reviews on this topic as plant proteins have highly variable amino acid compositions and may not stimulate MPS similarly to soy. A recent systematic review concluded that plant proteins were similar to animal protein for maintaining muscle mass, yet all trials in this review provided soy protein as the plant protein intervention. A careful approach must be taken to separate the effects of different plant proteins before it is possible to state that plant and animal proteins are comparable in terms of their effects on muscle health. Therefore, further synthesis of randomized controlled trial (RCT) data on this topic, with a greater diversity of plant proteins, is justified. A 2021 meta-analysis with a similar aim included a small number of non-soy interventions; however, certain methodological decisions reduce the confidence in the comparability of the plant and animal protein interventions and their effects on older adults as a discrete population with greater nutritional risk.^21^ For example, some RCTs had substantial differences in the gram weight of the plant compared with the animal protein intervention (up to 25.8 g in 1 RCT), and older adults were defined as being 50 years of age or older. This cutoff point may fail to capture the effects on those who are particularly vulnerable to sarcopenia, as results may be influenced by the inclusion of middle-aged adults who are likely to be more robust in terms of their physiological and molecular–biological functions. Furthermore, a precedent was set by the World Health Organization baseline report on healthy aging by defining older adults as aged 60 years or older,^22^ with consortia such as the Cochrane–Campbell Global Ageing Partnership following suit.^23^ Therefore, there is an argument for advancing aging research in line with this definition.

This systematic review aimed to synthesize available data from RCTs to evaluate the effect of plant vs animal protein on muscle mass, strength, physical performance, and sarcopenia status in young (<60 years) and older (≥60 years) adults. A second aim was to determine the influence of sex and intervention characteristics (eg, inclusion of RT and plant protein source) on the same outcomes.

## METHODS

**Figure 1.**
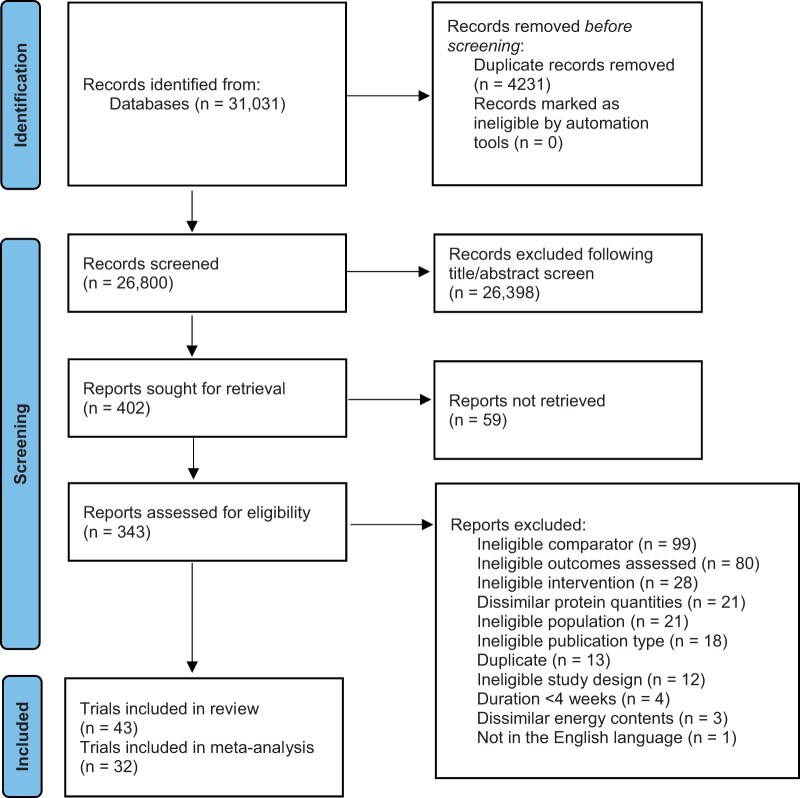
PRISMA © Flow Diagram Displaying the Number of Studies Retrieved, Screened, Assessed for Eligibility, and Excluded at Full-Text Review Stage, Including Reasons for Exclusion. Abbreviation: PRISMA, Preferred Reporting Items for Systematic Review and Meta-Analyses

This research was conducted according to the recommendations from the Preferred Reporting Items for Systematic Review and Meta-Analyses (PRISMA) 2020 checklist (Figure 1). The protocol was registered with PROSPERO (CRD42020188658) and has been published previously.^10^

### Eligibility Criteria

Studies were considered eligible if they were RCTs available as a full text (ie, not a conference abstract), published in the English language, and with a minimum intervention duration of 4 weeks (as significant increases in muscle mass and strength have previously been reported within this time frame).^24^^,^^25^ Adults aged 18 years or older who were not pregnant, breastfeeding, hospitalized, or bedridden were eligible. To maintain generalizability of findings, trials were ineligible if they were conducted in populations with a disease that affects the normal absorption/metabolism of, or requirements for, dietary protein, such as cancer, chronic kidney disease, or clinical malnutrition (Table 1). The trial was required to include at least 1 plant protein intervention and an animal protein comparator. The plant and animal protein interventions were required to be isocaloric and similar in protein content, defined as ±5 g for supplements, or the same percentage of protein as a total of dietary energy for whole-diet interventions. Trials that included a vitamin and/or mineral supplement and/or exercise, alongside the protein intervention, were permitted if these additional interventions were identical in the plant and animal protein arms. Trials were required to report 1 or more of the following outcomes: muscle mass (including lean/fat-free/muscle mass, muscle cross-sectional area, arm circumference), muscle strength, physical performance, and/or sarcopenia. The PICOS (Population, Intervention, Comparator, Outcome, and Study Design) criteria for eligibility are presented in Table 1.

**Table 1.** PICOS Criteria for Inclusion and Exclusion of Studies

Parameter	Inclusion criteria	Exclusion criteria
Population	Adults (≥18 y)	Pregnant or breastfeeding women; bedridden individuals; cancer, chronic kidney disease, clinically malnourished patients
Intervention	Plant protein (as a supplemented powder/drink, food, or diet)	A mixture of plant and animal proteins; plant proteins with additional nutrients added when the animal protein comparator did not receive the same additional nutrients
Comparator	Comparable weight of animal protein (+/–5 g) and with identical interventions otherwise (eg, exercise or vitamin/mineral supplements)	—
Outcomes	Muscle mass: magnetic resonance imaging (MRI), computed tomography (CT), dual-energy X-ray absorptiometry (DEXA), bioelectrical impedance (BIA), hydrostatic weighing, air displacement plethysmography, appropriate anthropometric measures Muscle strength: appendicular skeletal muscle strength measured by, eg, pinch strength, grip strength, 1 repetition maximum (1RM) with free weights or resistance machines, any other acceptable isometric or dynamic strength tests Physical performance: Timed-Up-and-Go (TUG) speed test, gait speed test, balance tests, Short-Performance Physical Battery (SPPB) test, repeated chair stands, any other functional test used in young or older adults to measure ability of muscle to perform a physical task Sarcopenia: using methods and cutoff points advised by expert bodies such as the European Working Group on Sarcopenia in Older People (EWGSOP) or Asian Working Group for Sarcopenia (AWGS)	—
Study design	Randomised–controlled trials	Non-randomised trials or observational studies

### Search Strategy, Screening, and Data Extraction

A comprehensive search strategy was developed by 3 reviewers (R.J.R.-M., M.C.M., C.T.M.) and included key terms such as “dietary protein”, “dairy”, “plant protein”, “muscle mass”, and “sarcopenia”. Original search terms and standardized medical subject headings (MeSH) were combined using Boolean operators. The search strategy has been published elsewhere.^10^ Trials published on or before June 15, 2023, were retrieved from 5 databases: Medline, Embase, Scopus, Web of Science, and Cochrane Central Register of Controlled Trials (CENTRAL). In addition, reference lists of key articles were manually searched. Authors were contacted in the case of unclear or missing data.

One reviewer (R.J.R.-M.) uploaded all titles to Rayyan (Qatar Computing Research Institute, Doha, Qatar) for screening. Titles and abstracts were independently screened against eligibility criteria by 2 reviewers (R.J.R.M., S.F.B.) and full-text articles were screened independently by a minimum of 2 reviewers (R.J.R.-M., S.F.B., N.A.W., and/or D.L.). Any discrepancies between reviewers relating to article eligibility were resolved by discussion and a consensus was reached. Data relating to trial population (mean age, sex, other descriptors as reported by studies, eg, overweight/obese, postmenopausal), intervention (duration, protein source, grams per day), comparator, and outcomes (measurement method, units, intervention effects) were extracted to Excel (Microsoft Excel for Mac, version 16.7; Microsoft Corporation, Redmond, WA, USA) using a standard template (this can be provided upon request).

### Quality Assessment

The quality of included RCTs was assessed independently by 2 of the reviewers (R.J.R.-M., S.F.B., N.A.W., D.L.) using the Cochrane Risk of Bias 2.0 (RoB2) tool for parallel-group or crossover RCTs.^26^ Reviewers determined whether each of 5 domains (randomization process, deviations from intended intervention, missing outcome data, measurement of outcomes, selective reporting) had a low or high risk of bias, or whether there were some concerns—for example, due to unclear or missing information regarding allocation concealment or method of randomization. Discrepancies between reviewers relating to risk of bias were resolved by discussion and consensus was reached.

### Data Synthesis

Characteristics of included RCTs were synthesized in a table with comprehensive data on interventions and comparators, population, duration, and outcome assessment. Data were synthesized quantitatively, where possible, or in narrative format otherwise.

### Statistical Analysis

Sufficient data meant that meta-analyses were possible for outcomes of muscle mass, muscle strength, and physical performance; however, there were no RCT data available to determine the effects of plant vs animal protein on the outcome of sarcopenia. The between-group mean difference and 95% CIs were calculated for absolute muscle mass, upper and lower body strength, and physical performance, and then pooled using random-effects models. The standardized mean difference (SMD) was calculated for each pooled analysis where different measurement methods were used or when different units of outcome measures were reported—for example, pounds (lb) and kilograms (kg) of muscle mass. The SMDs of 0.2, 0.5, and 0.8 were considered small, moderate, and large effect sizes, respectively.^27^

Standard formulas were used to convert reported effect estimates into the mean differences for meta-analysis, where applicable. For example, the 95% CI and sample size were used to estimate SD when not reported in the trial. If necessary, effect sizes were imputed using the prognostic method, which involved calculating the average variance reported in other included trials weighted by sample size. Previous research has confirmed this as a valid and accurate approach.^28^

The *I^2^* statistic was used to assess statistical heterogeneity, defined as “low” (0%–25%), “moderate” (25%–50%), “substantial” (50%–75%), and “high” (75%–100%).^29^ Risk of publication bias was assessed for pooled analyses with 10 or more RCTs using visual inspection of funnel plot asymmetry and Egger’s test.^30^

Where possible, subgroup analyses were conducted for the following: (1) older (≥60 years) and younger (<60 years) adults, (2) protein interventions with and without RT, and (3) protein supplements and whole-food/dietary interventions. Older adults were defined as those 60 years or over, as postabsorptive rates of myofibrillar MPS are slower in this age group than in those younger than 60 years^31^ and higher protein quality may be especially important in older age.

All statistical analyses were conducted using Review Manager version 5.4 (Cochrane Collaboration, Copenhagen, Denmark).

## RESULTS

The PRISMA flowchart for study selection is shown in Figure 1. The database searches generated 31 031 titles. Following removal of duplicates, 26 800 titles/abstracts were screened, and of these, 402 articles proceeded to full-text screening. Reasons for exclusion are also outlined in Figure 1.

### Study Characteristics

Forty-three RCTs met the eligibility criteria and are summarized in Table 2.^15–17^^,^^24^^,^^32–70^ Most of the studies (*n* = 23) were conducted in the United States,^16^^,^^17^^,^^32^^,^^33^^,^^42–45^^,^^47^^,^^48^^,^^50^^,^^51^^,^^55^^,^^56^^,^^58^^,^^61^^,^^63^^,^^64^^,^^66–68^^,^^70^^,^^71^ 3 studies were conducted in Canada,^49^^,^^53^^,^^62^ 2 were conducted in Australia,^36^^,^^41^ and Hong Kong,^59^^,^^60^ and 1 study was conducted in each of the following countries: Brazil,^40^ Chile,^69^ China,^38^ Denmark,^57^ France,^15^ Germany,^39^ Iran,^54^ Japan,^34^ Italy,^46^ Mexico,^52^ The Netherlands,^37^ Poland,^24^ and Sweden.^65^ The mean length of follow-up was 16 weeks, ranging from 4 to 104 weeks. Sample size ranged from 11 to 253 participants. Ten trials were conducted in older adults (mean age ≥60 years)^32^^,^^33^^,^^35–42^ and 32 trials were conducted in younger adults (<60 years),^15–17^^,^^24^^,^^43–70^ while 1 trial analyzed a group of younger and older adults separately.^34^

**Table 2.** Characteristics of Randomized Controlled Trials Comparing the Effects of Plant vs Animal Protein on Muscle Aging Outcomes (Muscle Mass, Strength, and Physical Performance)

Study (year) (country)	Population	**Study duration, weeks**	Age, y	No.	Protein source (g/d)	Post-intervention total daily protein, g/kg bw/d	Intervention characteristics	Intervention type	Exercise	Outcome (measurement method)
Trials included in meta-analysis (*n* = 32)										
Anderson et al (2007) (USA)^43^	Women with obesity	16	46.5 ± 8.4	17	Soy (91 g)	Not reported	Low-energy diet (1076-1195 kcal/d) consisting of soy or casein protein-based meal replacement shakes (3/d + entrée) plus fruit/vegetables Minimum total protein 91 g/d (15 g from entrée, ∼61.8 g from shakes, remainder from fruit/vegetables Aim of 8.4 MJ weekly physical activity	Meal replacement	Yes (PA)	Lean mass (ADP) Total lean tissue (DEXA)^a^^,^^b^
			44.0 ± 12.2	18	Casein (91 g)					
Baer et al (2011) (USA)^44^	Men and women with overweight or obesity	23	53 ± 9	25	Soy protein isolate (56 g)	1.4	Taken as a beverage twice a day, immediately before, during, or after breakfast and dinner Accompanied by daily vitamin and mineral supplement	Supplement	No	Lean mass (ADP) % Lean mass (ADP)
			49 ± 9	23	Whey protein concentrate (55 g)	1.4				
Basciani et al (2020) (Italy)^46^	Untrained men and women with insulin resistance and obesity	6	56.2 ± 6.1	16	Soy, pea, and cereal (90 g)	Not reported	Very-low-calorie ketogenic diets (VLCKD) (780 kcal/d). Five meals were consumed per day. Meals were provided in preassembled boxes. MNR = 14:46:40 Supplement containing vitamins, minerals, and omega-3 fatty acids was also provided. Thirty minutes of exercise 3 d/wk was encouraged but no formal exercise program was given and compliance not assessed.	Meal replacement	No	Lean mass (DEXA) % Lean mass (DEXA)^b^ Grip strength^c^
				16	Whey (90 g)					
				16	Meat, fish, and eggs (90 g)					
Barnard et al (2005) (USA)^45^	Overweight postmenopausal women	14	57.4	29	Vegan diet (15% of daily energy)	Not reported	Vegan diet (MNR = 75:15:10) consisting of vegetables, fruits, grains, and legumes, and devoid of animal products, added oils, avocados, olives, nuts, nut butters, and seeds. Omnivorous diet (MNR = 55:15:30) followed National Cholesterol Education Program Step II guidelines.	Diet	No	Lean mass (ADP)^a^
			55.6	30	Omnivorous diet (15% of daily energy)					
Beavers et al (2015) (USA)^32^	Older men and women with abdominal obesity	12	67.4 ± 4.5	12	Soy (28-36 g)	Not reported	Energy-restricted diets (-500 kcal/d) Lunch and dinner provided to participants; in addition, participants consumed 5 Medifast (Baltimore, MD, USA) meal-replacement shakes per day	Meal replacement	No	Lean mass, kg (DEXA) Lean mass, % (DEXA) Thigh muscle volume (CT) Grip strength Leg extension SPPB 400-m walk^c^
			69.5 ± 6.3	12	Whey and egg (28-36 g)					
Berger et al (2014) (USA)^47^	Healthy adolescent females	16	18.3 ± 0.4	62	Soy (20 g)	Not reported	Taken once a day as a beverage in place of usual breakfast	Meal replacement	No	Fat-free soft tissue mass (DEXA)^a^^,^^b^
			18.2 ± 0.4	58	Casein (20 g)					
DeNysschen et al (2009) (USA)^51^	Men with overweight and hyperlipidemia	12	38	9	Soy (26 g)	1.1 ± 0.3	Supplement mixed with 8 oz of water or fruit juice and taken within 60 min of training. On non-training days, the supplement was taken at a similar time. RT 3 d/wk	Supplement	Yes (RT)	Fat-free mass (Skinfold thickness; body weight minus fat mass) 11 Strength outcomes (1RM)^a^^,^^b^ squat, bench press, dumbbell bench press, shoulder press, triceps, bent-over-row, lunges, 1-arm row, upright row, fly, shrugs, lateral raises
				10	Whey (27 g)	1.2 ± 0.3				
Durkalec-Michalski et al (2022) (Poland)^24^	Young, trained CrossFit participants, men and women	4	31.0 ± 3.6	10	Vegan diet	Not reported	Individualized weight-maintenance diets, either vegan or traditional mixed diet Macronutrients in grams: carbohydrate: 4.5–5.5 g/kg bw/d; protein: 1.5–2.0 g/kg bw/d; fat: 0.8–1.5 g/kg bw/d) Progressive HIFT (CrossFit [Washington, DC, USA]) program with strength and aerobic components	Diet	Yes (HIFT)	Squat (70% 1RM)^b^ Deadlift (70% 1RM)^a^
			30.5 ± 3.0	10	Traditional mixed diet					
Evans et al (2007) (USA)^33^	Postmenopausal women	39	63.5 ± 4.8	10	Soy protein isolate (26 g)	Not reported	Taken as a beverage once daily. Participants were advised to use as a protein substitute rather than supplement to maintain energy balance. Protein beverages also included 900 mg calcium and 125 IU vitamin D. Half of participants engaged in supervised endurance exercise on 3 d/wk.	Substitute	No	Lean mass (DEXA)
			62.8 ± 5.3	12	Milk protein isolate (26 g)					
			62.5 ± 5.3	11	Soy protein isolate (26 g)				Yes	
			59.7 ± 5.2	12	Milk protein isolate (26 g)					
Gonzáles-Salazar et al (2021) (Mexico)^52^	Men and women with obesity and insulin resistance	4	40.6 ± 12.5	18	Normal protein diet (19% total energy) with 60% protein from animal sources	Not reported	Menus provided for hypocaloric diet (1800 kcal/d) and either normal protein or high protein diets with predominance of animal or plant protein.	Diet	No	Skeletal muscle mass % (BIA)^a^^,^^b^ Fat-free mass % (BIA)^a^^,^^b^ Grip strength
			39.3 ± 11.4	18	Normal protein diet (19% total energy) with 60% protein from plant sources					
			37.7 ± 8.4	19	High protein diet (29.5% total energy) with 60% protein from animal sources					
			35.7 ± 9.9	20	High protein diet (29% total energy) with 60% protein from plant sources					
Hartman et al (2007) (Canada)^53^	Healthy, untrained young men	12	18-30	19	Fat-free soy protein drink (17.5 g)	Not reported	Participants consumed 500 mL of soy or milk beverages immediately after training, and again 1 h after training. A total of 60 RT sessions were completed across 12 wk (5 d/wk).	Supplement	Yes (RT)	Fat- and bone-free mass (DEXA)^a^^,^^b^ 11 Strength outcomes (1RM): incline leg press, knee extension, hamstring curl, military press, bench press, triceps push down, front pectoral fly, lateral pull down, wide grip seated row, standing biceps curl, rear deltoid fly
				18	Fat-free milk (17.5 g)					
Haub et al (2002) (USA)^35^	Older men	12	67 ± 6	11	Soy TVP products	1.15 ± 0.1	Self-selected lacto-ovo- vegetarian diet supplemented with 0.6 g/kg bw/d of either soy-based TVP products or beef RT 3 d/wk	Diet	Yes (RT)	Fat-free mass (ADP) Vastus lateralis CSA (CT) Leg extension (1RM)^a^^,^^b^ Leg flexion (1RM)^a^^,^^b^ Leg press (1RM)^a^^,^^b^ Chest press (1RM)^a^^,^^b^ Arm pull (1RM)^a^^,^^b^
			63 ± 3	10	Beef	1.03 ± 0.3				
Hill et al (2015) (USA)^55^	Men and women with overweight or obesity and metabolic syndrome	23	45.3 ± 6.7	21	Plant protein (two-thirds total protein in diet)	Not reported	The “M-DASH” diet—two-thirds of total protein from plant sources (pulses, grains, soy, nuts, and seeds)—or the “BOLD” diet—two-thirds protein from animal sources (lean beef, chicken, eggs, dairy) MNR = 55:18:27 Energy-restricted diets (-500 kcal/d) from months 4–6) Participants provided with a pedometer and asked to reach 10 000 steps/d by the end of the weight-loss phase (week 11)	Diet	Yes (walking)	Body lean mass (DEXA)^a^^,^^b^ Abdominal lean mass (DEXA)^a^^,^^b^
			46.2 ± 9.4	20	Animal protein (two-thirds total protein in diet)					
Jadczak et al (2021) (Australia)^36^	Prefrail and frail older adults	24	73.2 ± 6.6	30	Rice (40 g)	1.4 ± 0.4	20 g of rice or whey protein taken twice daily as a powder mixed with 150 mL water. On training days, participants consumed 1 sachet within 1 h of exercise and the other in-between main meals. On non-training days, participants consumed 1 sachet as a mid-morning snack and the other as a mid-afternoon or evening snack. Five exercise sessions per week: one 60-min supervised group exercise class, two 45-min home-based classes, and walking twice per week for at least 30 min.	Supplement	Yes (multimodal)	Muscle mass (BIA) Grip strength^a^^,^^b^ Gait speed Timed Up-and-Go^a^^,^^b^ SPPB^a^^,^^b^
			73.5 ± 7.2	23	Whey (40 g)	1.3 ± 0.5				
Joy et al (2013) (USA)^17^	Healthy, trained young men	8	21.3 ± 1.9	12	Rice protein (48 g)	Not reported	Powder dissolved in 500 mL water and taken once per day, on training days only. Ingestion was supervised by a researcher. Diets also macronutrient controlled (MNR: 50:25:25). RT program that aimed to train main muscle groups 2 d/wk.	Supplement	Yes (RT)	Lean body mass (DEXA)^a^^,^^b^ Bicep muscle thickness (ultrasound)^a^^,^^b^ Quadriceps muscle thickness (ultrasound)^a^^,^^b^ Bench press (1RM)^a^^,^^b^ Leg press (1RM)^a^^,^^b^
				12	Whey protein (48 g)					
Kjølbæk et al (2017) (Denmark)^57^	Men and women overweight or obesity	24	40.0 ± 10.7	36	Soy protein (45 g)	1.57 ± 0.36	Taken 3 times daily as a part of main meals; supplement accounted for 10%-15% of daily energy	Supplement	No	Lean body mass (DEXA)^a^^,^^b^
				39	Whey protein (45 g)	1.66 ± 0.36				
Kok et al (2006) (Netherlands)^37^	Postmenopausal women	52	66.6 ± 4.8	75	Soy protein (26 g)	Not reported	Powder taken once daily Accompanied by supplements of vitamins B_2_, B_6_, and B_12_; folic acid, vitamin D; and calcium	Supplement	No	Grip strength SPPB
			66.8 ± 4.7	77	Casein protein (26 g)					
Li et al (2016) (USA)^58^	Men and women with obesity	12	56 ± 4	17	Plant protein (soy, legumes) diet	Not reported	Energy-restricted (-750 kcal/d) lacto-ovo-vegetarian diet with soy and legumes as predominant protein source, or an omnivorous diet with beef and pork as predominant protein source Protein content progressively increased across 12 wk (weeks 1–4: MNR = 65:10:25; weeks 5–8: MNR = 55:20:25; weeks 9–12: MNR = 45:30:25) Diets supplemented with daily multivitamin/mineral and twice-daily calcium (400 mg) and vitamin D (500 IU) supplements	Diet	No	Fat-free mass (ADP) Percentage fat-free mass
			51 ± 2	17	Animal protein (beef, pork) diet					
Li et al (2021) (China)^38^	Older adults with low lean mass	24	69 ± 4	31	Soy protein (18 g)	1.51 ± 0.41	8.8 g of soy protein or 8 g whey protein taken as a powder, mixed with 100 mL of warm water, twice per day	Supplement	No	Lean mass (DEXA) Grip strength Gait speed Chair stand SPPB
			71 ± 4	31	Whey protein (16 g)	1.39 ± 0.24				
Liu et al (2010) (Hong Kong)^59^	Postmenopausal women with mild hyperglycemia	26	56.4 ± 4.7	57	Soy protein (15 g)	Not reported	Taken as a powder mixed with 300 mL water or another beverage once daily	Supplement	No	Fat-free mass (BIA)
			56.0 ± 4.4	57	Milk protein with isoflavones (15 g)^d^					
			55.9 ± 3.8	50	Milk protein without isoflavones (15 g)					
Liu et al (2013) (Hong Kong)^60^	Postmenopausal women with prehypertension	26	57.6 ± 5.3	85	Soy protein (13 g)	Not reported	Participants advised to mix powder with 300 mL water or other beverage and partially replace meal or snack with protein shake	Substitute	No	Fat-free mass (BIA)
			57.7 ± 5.0	87	Milk protein with daidzein (13 g)^d^					
			58.5 ± 4.7	81	Milk protein without daidzein (13 g)					
Lukaszuk et al (2007) (USA)^61^	Premenopausal women with overweight but otherwise healthy	8	33.71 ± 6.32	7	Soy milk (25 g)	Not reported	Participants consumed 720 mL of soy or cow milk per day as part of a –500-kcal/d energy-restricted diet. Dietary counseling and meal plans were provided by a registered dietitian.	Supplement	No	Fat-free mass (ADP)^a^^,^^b^
			29.43 ± 11.03	7	Cow milk (25 g)					
Markova et al (2016) (Germany)^39^	Men and women with type 2 diabetes and NAFLD	6	63.7 ± 1.5	19	Plant protein (legumes) diet	Not reported	High protein diets (MNR = 40:30:30) with protein mainly from legumes, or an isocaloric and isonitrogenous diet with protein mainly from animal sources including meat and dairy	Diet	No	Fat-free mass % (ADP)^b^
			65.0 ± 1.4	18	Animal protein (meat and dairy) diet					
McBreairty et al (2020) (Canada)^62^	Women with polycystic ovarian syndrome (PCOS)	16	26.6 ± 5.0	29	Pulse-based diet	Not reported	In the pulse-based diet, 2 daily meals were provided containing approximately 150 g dry weight of peas, lentils, chickpeas, and beans. Aerobic exercise for 45 min/d, 5 d/wk (3 supervised at research center)	Diet	Yes (aerobic)	Lean mass (DEXA)
			26.8 ± 4.5	31	Therapeutic Lifestyle Changes (TLC) diet with chicken and fish advised as protein sources					
Moeller et al (2003) (USA)^63^	Perimenopausal women	24	50.2^2^	24	Soy protein with isoflavones (40 g)^d^	Not reported	Half taken in the form of a powder mixed with food or drink and half as a “jumbo muffin” each day. Intervention provided 500 kcal/d and therefore was considered a meal replacement rather than supplement. Multivitamin/multimineral also supplied to participants.	Meal replacement	No	Total lean mass (DEXA)^a^^,^^b^ Lean mass at waist region^a^^,^^b^ Lean mass at hip region ^a^^,^^b^ Lean mass at thigh region ^a^^,^^b^
			50.9^2^	24	Soy protein without isoflavones (40 g)					
			49.4^2^	21	Whey protein (40 g)					
Moon et al (2020) (USA)^64^	Resistance-trained men	8	32.8 ± 6.7	12	Rice protein (24 g)	1.43 ± 0.7	24 g/d protein ingested within 60 min of workout completion (on training days) or within 60 min of sleeping (on non-training days) RT 4 d/wk (2 d/wk upper body and 2 d/wk lower body)	Supplement	Yes (RT)	Fat-free mass (DEXA) ^a^^,^^b^ Lean mass (DEXA) ^a^^,^^b^ Bench press (1RM)^a^^,^^b^ Leg press (1RM)^a^^,^^b^
				12	Whey protein (24 g)	1.76 ± 0.6				
Pettersson et al (2021) (Sweden)^65^	Untrained men with overweight or obesity	6	29.3 ± 5.8	8	Oat and potato protein (20 g)	Not reported	A test drink with oat and potato protein (10 g protein) or milk protein (9 g protein) was taken once 15 min before exercising and another immediately after exercise. Supervised endurance training (treadmill, rowing machine, or exercise bike)—17 sessions in 6 wk, each lasting 60-75 min	Supplement	Yes (aerobic)	Lean mass (DEXA) ^a^^,^^b^
			28.2 ± 5.5	10	Milk protein (18 g)					
Roschel et al (2021) (Brazil)^40^	Prefrail or frail older women	16	72 ± 6	22	Soy protein (30 g)	1.26 ± 0.32	15 g soy or whey protein taken twice a day mixed with 150 mL water, once immediately after breakfast and once immediately after dinner Supervised, one-on-one RT 2 times/wk	Supplement	Yes (RT)	Lean mass (DEXA) ^a^^,^^b^ Vastus lateralis CSA (US) ^a^^,^^b^ Grip strength Leg press (1RM)^a^^,^^b^ Bench Press (1RM)^a^^,^^b^ Timed Up-and-Go Timed stands^a^^,^^b^
			72 ± 6	22	Whey protein (30 g)	1.19 ± 0.32				
Thomson et al (2016) (Australia)^41^	Healthy older men and women	12	61.7 ± 8.3	26	Soy protein (27 g)	1.45 ± 0.14	Taken once per day as a beverage Overall diet designed to be low-fat (30% fat, <8% saturated fat) and to maintain energy balance Whole-body RT program 3 d/wk	Supplement	Yes (RT)	Lean mass (DEXA)^a^^,^^b^ Grip strength ^a^^,^^b^ Leg press (8RM) ^a^^,^^b^^,^^c^ Chest press (8RM) ^a^^,^^b^ Knee extension (8RM) ^a^^,^^b^ Lateral pull down (8RM) ^a^^,^^b^ Leg curl (8RM) ^a^^,^^b^ Total (8RM) ^a^^,^^b^^,^^c^ 6-Minute Walk Test ^a^^,^^b^
			61.3 ± 6.9	34	Milk protein (27 g)	1.42 ± 0.14				
Volek et al (2013) (USA)^67^	Untrained younger men and women	39	24.0 ± 2.9	22	Soy protein (20 g)	1.35 ± 0.13	Powder mixed with 240 mL water and taken once daily, either at breakfast or postexercise on training days Supplements also contained 200 mg para-aminobenzoic acid (PABA) Protein goal of 1.0-1.2 g/kg bw/d (before supplementation); energy needs determined by RMR + activity Supervised whole-body RT program (96 workouts in total)	Supplement	Yes (RT)	Lean body mass (DEXA) ^a^^,^^b^^,^^c^ Squat (1RM) ^a^^,^^b^ Bench press (1RM) ^a^^,^^b^
			22.8 ± 3.7	19	Whey protein (22 g)	1.39 ± 0.18				
Vupadhyayula et al (2009) (USA)^42^	Postmenopausal women	104	63.63 ± 0.56	20	Soy protein without isoflavones (25 g)	Not reported	Powder added to water or other beverage and taken once per day Supplements also included 625 IU vitamin A, 1 µg vitamin B_12_, 55 µg folate, 125 IU vitamin D, 500 mg calcium, 2 mg iron, 90 mg magnesium, 500 mg phosphorus, 100 mg potassium, and 190 mg sodium	Supplement	No	Lean body mass (DEXA) Grip strength, nondominant hand Grip strength, dominant hand Knee extension (ID) Timed Up-and-Go Timed stands
			63.42 ± 0.56	30	Soy protein with isoflavones (25 g)^d^					
			63.76 ± 0.52	35	Casein whey protein blend (25 g)					
Zbinden-Foncea et al (2023) (Chile)^69^	Untrained young men	8	22.4 ± 3.1	6	Chia seed protein (20 g)	1.8 ± 0.7	Chia flour or whey protein drinks consumed within 10 min of exercise Supervised progressive RT program, 3 times/wk	Supplement	Yes (RT)	Fat-free mass (DEXA) ^a^^,^^b^ Chest press (1RM) ^a^^,^^b^ Shoulder press (1RM) ^a^^,^^b^ Lateral pull down (1RM) ^a^^,^^b^ Seated row (1RM) ^a^^,^^b^ Leg press (1RM) ^a^^,^^b^ Leg extension (1RM) ^a^^,^^b^
				6	Whey protein (23 g)	1.6 ± 0.5				
Trials included in narrative review (*n* = 11)										
Babault et al (2015) (France)^15^	Healthy, untrained young men	12	22.0 ± 3.5	47	Pea protein isolate (50 g)	Not reported	Half of powdered protein taken twice daily (in morning and post-training/in afternoon), mixed with 300 mL water. Upper-body RT, 3 d/wk	Supplement	Yes (RT)	Bicep muscle thickness (ultrasound) ^a^^,^^b^ Right arm circumference, at rest (tape) ^a^^,^^b^ Right arm circumference, contracted (tape) ^a^^,^^b^ Maximal voluntary torque (ID) ^a^^,^^b^ Arm curl (1RM) ^a^^,^^b^
			22.1 ± 3.6	46	Whey protein concentrate (50 g)					
Banaszek et al (2019) (USA)^16^	Healthy, trained men and women	8	Women: 38.9 ± 10.9 Men: 38.6 ± 10.7	8	Pea (49 g)	1.7 ± 0.4	Supplement mixed with 350 mL of water and consumed before and after training, or on non-training days, half in the morning and half in the evening HIFT (CrossFit [Washington, DC, USA]) 4 d/wk	Supplement	Yes (HIFT)	Rectus femoris thickness (US) Vastus lateralis thickness (US) Squat (1RM) ^a^^,^^b^ Deadlift (1RM) ^a^^,^^b^
				7	Whey (49 g)	1.8 ± 0.3				
Hashimoto et al (2015) (Japan)^34^	Highly active men and women in mid- to later life	4	59 ± 2	10	Soy (8 g)	Not reported	Taken once daily as a powder mixed into food or drink of participant’s choice.	Supplement	No	Quadriceps volume (MRI) Quadriceps strength
			62 ± 2	10	Casein (8 g)					
Hashimoto et al (2015) (Japan)^34^	Young, sedentary males	4	23 ± 0	4	Soy (8 g)		Taken once daily as a powder mixed into food or drink of participant’s choice	Supplement	No	Quadriceps volume (MRI)^a^^,^^c^ Quadriceps strength^a^^,^^c^
			26 ± 2	7	Casein (8 g)					
Brown et al (2004) (USA)^48^	Healthy, trained young men	9	21.67 ± 0.24	9	Soy (33 g)	Not reported	Three protein bars consumed per day, each providing 11 g protein RT program	Supplement	Yes (RT)	Lean body mass (HW) ^a^^,^^b^
			20.36 ± 0.34	9	Whey (33 g)					
Candow et al (2006) (Canada)^49^	Untrained young men and women	6	22.5 ± 6.0	9	Soy (1.2 g/kg body mass)	1.8 ± 1.4	Taken dissolved in water before training, after training, and before bed. On non-training days, participants consumed the supplement in 3 equal doses across the day. Supervised RT program 3 d/wk	Supplement	Yes (RT)	Lean mass (DEXA) ^a^^,^^b^ Squat (1RM) ^a^^,^^b^ Bench press (1RM) ^a^^,^^b^
			24.0 ± 6.0	9	Whey (1.2 g/kg body mass)	1.9 ± 1.3				
Christie et al (2010) (USA)^50^	Postmenopausal women with obesity	12	54.4 ± 3.3	17	Soy (20 g)	Not reported	Taken as a beverage, half with breakfast and half with dinner	Supplement	No	Lean mass (DEXA)
			53.3 ± 4.9	16	Casein (20 g)					
Hassanzadeh-Rostami et al (2019) (Iran)^54^	Men and women with type 2 diabetes	8	57.1 ± 7.3	21	Soybeans	1.14 ± 0.22	Identical diets but with 2 servings 3 d/wk of red meat (1 serving = 30 g) or soybeans (1 serving = 0.5 cup) MNR = 55:15:30	Food	No	% Muscle mass (BIA)
			59.6 ± 6.0	20	Non-soy legumes	1.02 ± 0.16				
			56.1 ± 7.2	23	Red meat	1.16 ± 0.21				
Kalman et al (2007) (USA)^56^	Healthy young men	12	31.6 ± 5.9	5	Soy protein concentrate (50 g)	Not reported	Participants asked to dissolve powder in 10-12 oz water and ingest half within 1 h of training and remaining half later in the day. RT 3 d/wk	Supplement	Yes (RT)	Lean body mass (DEXA)
			30.3 ± 8.1	5	Soy protein isolate (50 g)					
			31.4 ± 5.1	5	Whey protein blend (50 g)					
Sites et al (2007) (USA)^66^	Postmenopausal women	12	55.0 ± 5.4	9	Soy protein (20 g)	Not reported	Powder mixed with water; half taken with breakfast and half before bed	Supplement	No	Fat-free mass (DEXA)
			57.8 ± 4.3	6	Casein protein (20 g)					
Wilson et al (2022) (USA)^68^	Men and women with overweight or obesity	8	43.0 ± 12.3	8	Meals based on green lentils (18 g)	Not reported	All participants were provided with 5 midday meals each week which contained either 600 g total of green lentils or meals with chicken and turkey instead of lentils.	Diet	No	Skeletal muscle mass percentage (BIA)
			38.8 ± 10.9	11	Meals with chicken and turkey instead of lentils (23 g)					
Zemel et al (2010) (USA)^70^	Men and women with overweight or obesity	4 (crossover)	31.0 ± 10.3	10	Soy protein (30 g)	Not reported	Taken as a drink at 3 separate times across the day (2 at research center and 1 off-site) Energy-balanced diet (1.2-1.4 RMR) based on indirect calorimetry	Supplement	No	Lean mass (DEXA)
				10	Milk protein (30 g)					

a Significant change from baseline in plant group.

b Significant change from baseline in animal protein group.

c Significant difference between groups.

d The group used as the comparator in analysis.

Abbreviations: ADP, air displacement plethysmography; BIA, bioelectrical impedance analysis; bw, body weight; CSA, cross-sectional area; CT, computed tomography; DEXA, dual-energy X-ray absorptiometry; HIFT, high-intensity functional training; HW, hydrostatic weighing; ID, isokinetic dynamometry; MNR, macronutrient ratio (presented as carbohydrate:protein:fat); MRI, magnetic resonance imaging; NAFLD, nonalcoholic fatty liver disease; RM, repetition maximum; RMR, resting metabolic rate; RT, resistance training; PA, Physical activity; SPPB, Short-Performance Physical Battery; TVP, textured vegetable protein; US, ultrasound.

### Intervention Characteristics

Interventions are described in detail in Table 2. In brief, 26 RCTs (60%) tested the effects of plant protein powder supplements, including soy,^34^^,^^37^^,^^38^^,^^40–42^^,^^44^^,^^48–51^^,^^53^^,^^56^^,^^57^^,^^59^^,^^61^^,^^66^^,^^67^^,^^70^ rice,^17^^,^^36^^,^^64^ pea,^15^^,^^16^ oat and potato,^65^ and chia seed proteins.^69^ All protein supplement trials used milk protein as the animal comparator. Seven RCTs (17%) evaluated protein meal replacements or substitutes.^32^^,^^33^^,^^43^^,^^46^^,^^47^^,^^60^^,^^63^ A further 10 RCTs (23%) were dietary interventions, such as a vegan diet,^24^^,^^45^ a high–plant protein diet,^52^^,^^55^ or with plant protein food sources.^35^^,^^39^^,^^54^^,^^58^^,^^62^^,^^68^ The animal comparators in these dietary RCTs varied from meat, fish, and dairy to omnivorous diets. Seven trials in overweight/obese populations featured an energy restriction with weight loss being the primary outcome.^32^^,^^43^^,^^46^^,^^52^^,^^55^^,^^58^^,^^61^ Sixteen out of 43 trials (37%) included RT alongside the plant or animal protein interventions.^15–17^^,^^24^^,^^35^^,^^36^^,^^40^^,^^41^^,^^48^^,^^49^^,^^51^^,^^53^^,^^56^^,^^64^^,^^67^^,^^69^

### Outcomes

As shown in Table 2, 41 RCTs (95%) assessed muscle mass using a range of methods, primarily with dual-energy X-ray absorptiometry (DEXA),^17^^,^^32^^,^^33^^,^^38^^,^^40–43^^,^^46^^,^^47^^,^^49^^,^^50^^,^^53^^,^^55–57^^,^^62–67^^,^^69^^,^^70^ air displacement plethysmography (ADP),^35^^,^^39^^,^^43–45^^,^^58^^,^^61^ and bioelectrical impedance analysis (BIA).^36^^,^^52^^,^^54^^,^^59^^,^^60^^,^^68^ A total of 21 RCTs assessed 15 different muscle strength endpoints—for example, bench press,^17^^,^^35^^,^^40^^,^^49^^,^^51^^,^^53^^,^^64^^,^^67^^,^^69^ hand-grip strength,^32^^,^^36–38^^,^^40–42^^,^^46^^,^^52^ and leg extension.^32^^,^^34^^,^^35^^,^^41^^,^^42^^,^^53^^,^^69^ Six different methods of assessing physical performance were used across the 7 RCTs (16%) reporting this outcome (Table 2).^32^^,^^36–38^^,^^40–42^ No trials were identified that examined the effects of plant vs animal protein on sarcopenia status.

### Assessment of Risk of Bias at the Individual Study Level

**Figure 2.**
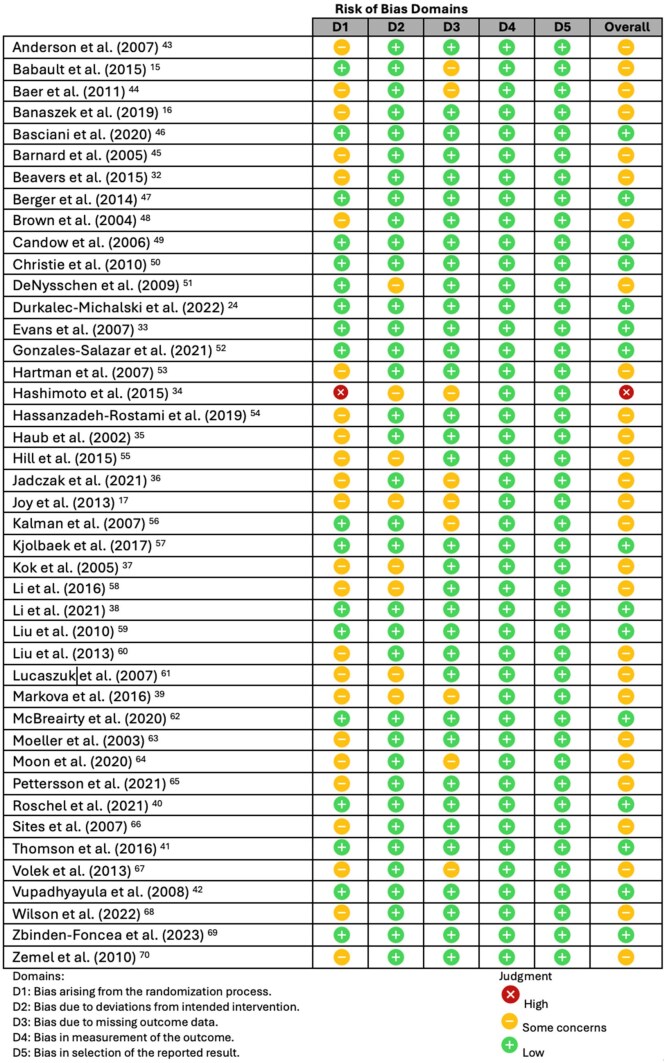
Quality Assessment of 43 Included Trials Using Cochrane Risk of Bias 2.0 (RoB2)

Figure 2 15–17 ^,^ ^24^ ^,^ 32–70 displays the risk of bias in the 43 included trials. Fifteen trials (35%) were determined to have a low risk of bias overall,^24^^,^^33^^,^^38^^,^^40–42^^,^^46^^,^^47^^,^^49^^,^^52^^,^^57^^,^^59^^,^^62^^,^^69^^,^^72^ there were concerns about risk of bias in 27 trials (63%),^15–17^^,^^32^^,^^35–37^^,^^39^^,^^43–45^^,^^48^^,^^51^^,^^53–56^^,^^58^^,^^60^^,^^61^^,^^63–68^^,^^70^ and 1 trial (2%) had a high risk of bias due to a substantial lack of information on randomization procedures and baseline imbalances.^34^ Compliance was good to excellent in most trials, with supervised supplement ingestion and RT, monitoring of empty supplement packets, food diaries, and objective biomarkers used as compliance assessment methods (Table S1).^15–17^^,^^24^^,^^32–70^

### Meta-analysis of the Effects of Plant vs Animal Protein on Muscle Aging

**Figure 3.**
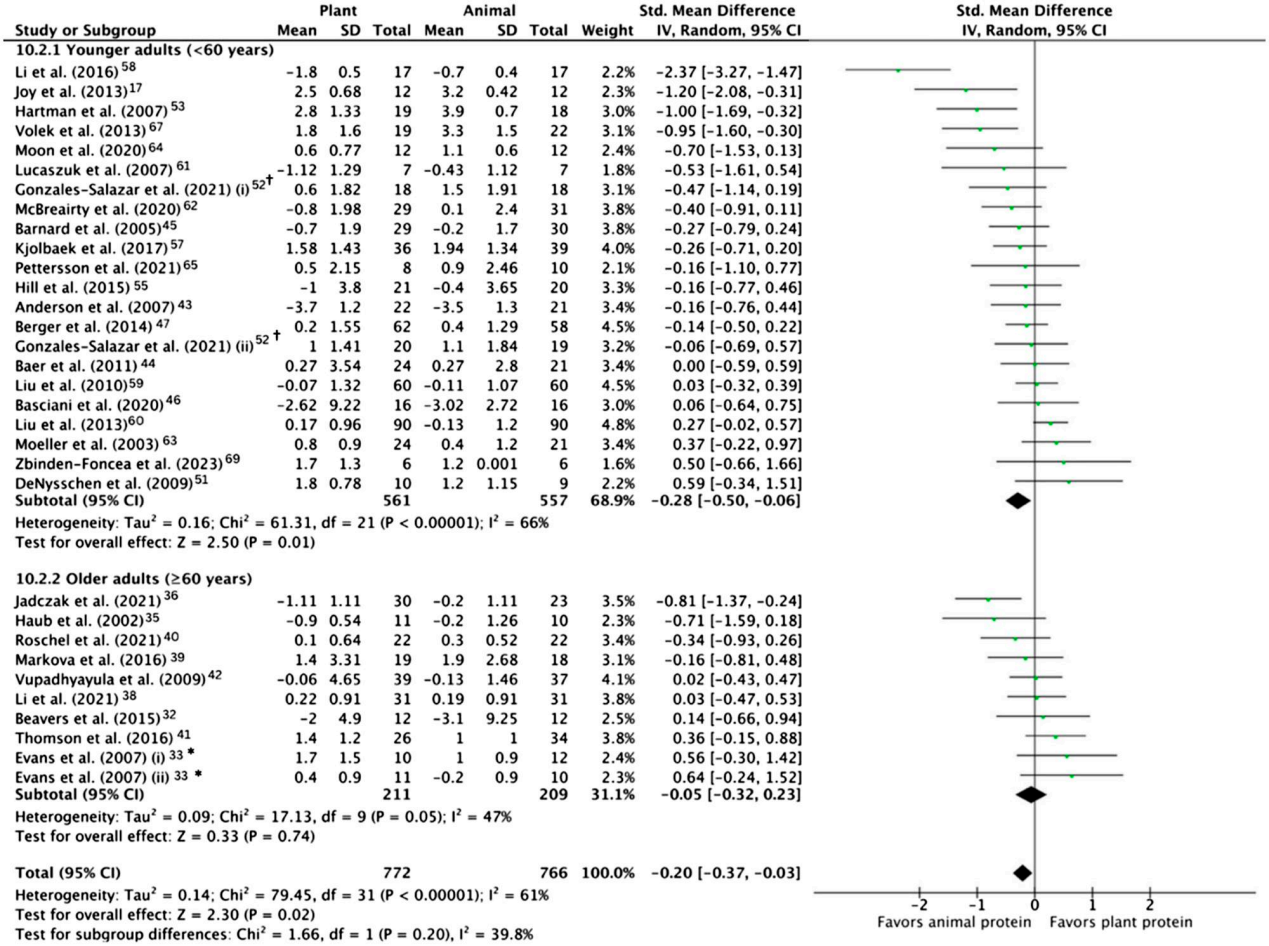
Meta-analysis of Change in Absolute Muscle Mass in Response to Plant vs Animal Protein Intervention, Overall and in Younger (<60 y) and Older (≥60 y) Adults. *Evans et al^33^ (i) denotes the groups who received the protein intervention alone and (ii) denotes those who received protein and exercise interventions. ^†^Gonzáles-Salazar et al^52^ (i) denotes the groups who received a normal protein diet (19% of daily energy) and (ii) denotes those who received a high protein diet (29% of daily energy). Abbreviations: IV, inverse variance; Std, standardized

**Figure 4.**
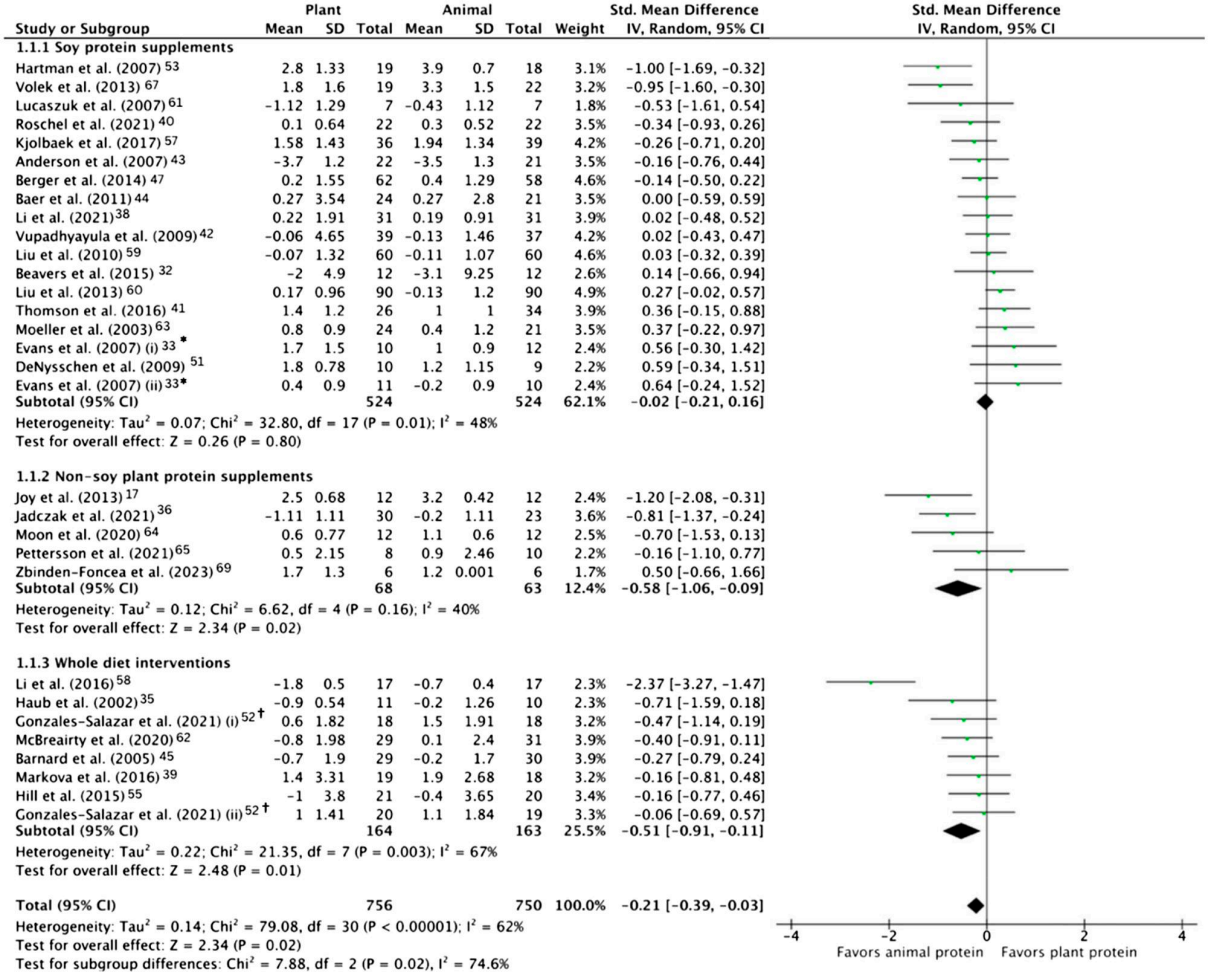
Subgroup Analysis of Change in Absolute Muscle Mass in Response to Plant vs Animal Protein Interventions, Grouped by Plant Protein Source. One trial was excluded from subgroup analysis because the plant protein supplement was a combination of soy and non-soy proteins.^46^ *Evans et al^33^ (i) denotes the groups who received the protein intervention alone and (ii) denotes those who received protein and exercise interventions. ^†^Gonzáles-Salazar et al^52^ (i) denotes the groups who received a normal protein diet (19% of daily energy) and (ii) denotes those who received a high protein diet (29% of daily energy). Abbreviations: IV, inverse variance; Std, standardized

**Figure 5.**
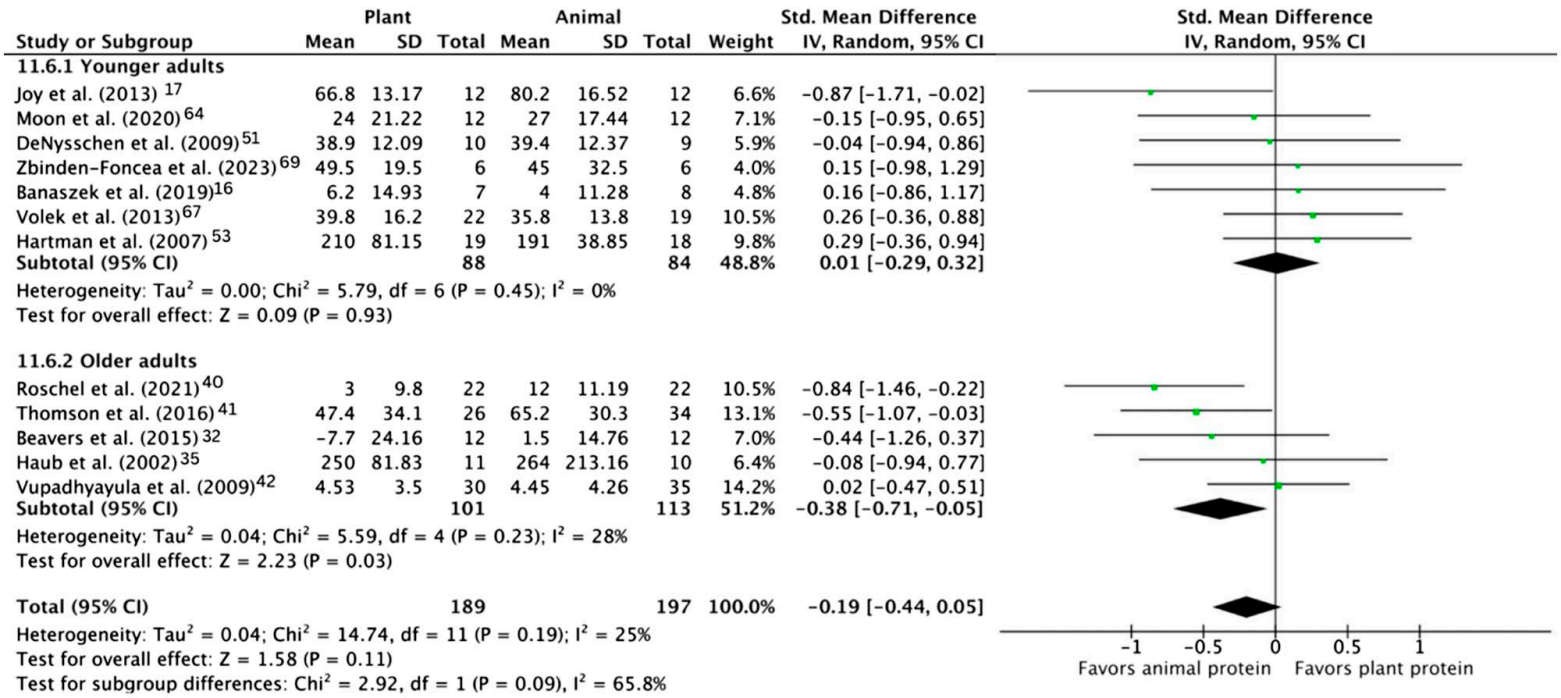
Meta-analysis of Change in Lower Body Strength in Response to Plant vs Animal Protein Interventions, Overall and in Younger (<60 y) and Older (≥60 y) Adults. Abbreviations: IV, inverse variance; Std, standardized

The pooled effect of plant vs animal protein interventions on muscle aging endpoints is shown in Figures  3–5 and described below.

#### Effects on Muscle Mass

Thirty RCTs involving 1538 participants (772 plant protein intervention, 766 animal protein comparator) reported muscle mass endpoints suitable for meta-analysis. As shown in Figure 3,^17^^,^^32^^,^^33^^,^^35^^,^^36^^,^^38–47^^,^^51–53^^,^^55^^,^^57–65^^,^^67^^,^^69^ the pooled analysis of all 30 RCTs indicated a small superior effect of animal protein over plant protein on muscle mass (SMD = −0.20; 95% CI: −0.37, −0.03; *P* = .02), with substantial between-study heterogeneity (*I^2^* = 62%).

#### Subgroup Analysis by Age, Sex, Protein Source, and Inclusion of RT Intervention

In subgroup analyses of different age groups (presented in Figure 3), the superior effect of animal protein on muscle mass was stronger in younger (*n* = 21 RCTs; SMD = -0.28; 95% CI: −0.50, −0.06; *P* = .01) than older adults (*n* = 9 RCTs; SMD = -0.05; 95% CI: −0.32, 0.23; *P* = .74). Subgroup analysis by sex found a small but nonsignificant superior effect of animal protein in men (SMD = -0.44; 95% CI: −0.95, 0.06; *P* = .09) and no difference in women (SMD = 0.00; 95% CI: −0.17, 0.17; *P* = .99).

Figure 4 ^17^ ^,^ ^32^ ^,33,35,36,^ 38–47 ^,^ 51–53 ^,55,^ 57–65 ^,67,69^ displays subgroup analysis for the effect of different protein sources on muscle mass. Pooled data from 17 RCTs indicated no difference in muscle mass between soy protein and milk protein. Further subgroup analysis by age (<60 years, ≥60 years) and sex revealed no significant differences between soy and milk protein (data not shown). In a smaller number of trials (*n* = 5), animal protein had a more favorable pooled effect on muscle mass compared with non-soy sources of plant protein (SMD = -0.58; 95% CI: −1.06, −0.09; *P* = .02). Furthermore, in 7 diet trials involving 327 participants, following an isocaloric animal protein diet resulted in greater beneficial effects on muscle mass compared with a plant diet (SMD = -0.51; 95% CI: −0.91, −0.11; *P* = .01).

The superior effect of animal protein was stronger in trials with RT (SMD = -0.45; 95% CI: −0.85, −0.05; *P* = .03), than trials without RT (SMD = -0.10; 95% CI: −0.27, 0.08; *P* = .27) (Table 3).^17^^,^^32^^,^^33^^,^^35^^,^^36^^,^^38–47^^,^^51–53^^,^^55^^,^^57–65^^,^^67^^,^^69^ This pooled effect difference in trials with RT was greater in younger (SMD = -0.54; 95% CI: −1.10, 0.03; *P* = .06) than in older (SMD = -0.34; 95% CI: −0.91, −0.24; *P* = .25) adults (Table 3).

**Table 3.** Meta-analysis of Trials Comparing the Effect of Plant vs Animal Protein on Muscle Mass, With or Without Resistance Training, by Age Group (Young [<60 y] and Older [≥60 y] Adults)

Trial characteristics	No. of trials, with citations	Pooled sample size, *n*		Effect size [95% CI]	*P*	*I^2^*
		Plant	Animal			
Without resistance training	20^32^^,^^33^^,^^38^^,^^39^^,^^42–47^^,^^52^^,^^55^^,^^57–63^^,^^65^	605	598	−0.10 [−0.27, 0.08]	.27	52%
<60 y	15^43–47^^,^^52^^,^^55^^,^^57–63^^,^^65^	483	478	−0.19 [−0.40, 0.03]	.09	61%
≥60 y	5^32^^,^^33^^,^^38^^,^^39^^,^^42^	122	120	0.11 [−0.15, 0.36]	.41	0%
With resistance training	10^17^^,^^35^^,^^36^^,^^40^^,^^41^^,^^51^^,^^53^^,^^64^^,^^67^^,^^69^	167	168	−0.45 [−0.85, -0.05]	.03	66%
<60 y	6^17^^,^^51^^,^^53^^,^^64^^,^^67^^,^^69^	78	79	−0.54 [−1.10, 0.03]	.06	64%
≥60 y	4^35^^,^^36^^,^^40^^,^^41^	89	89	−0.34 [−0.91, 0.24]	.25	71%

### Effects on Muscle Strength

#### Lower Body Strength

Figure 5 ^16^ ^,^ ^17^ ^,32,35,^ 40–42 ^,51,53,64,67,69,73^ displays meta-analysis of 11 trials that reported lower body strength as an outcome, measured by squat, leg press, or leg extension. Animal protein was significantly beneficial compared with plant protein (SMD = -0.38; 95% CI: −0.71, −0.05; *P* = .03) in older adults but not in younger adults (SMD = 0.01; 95% CI: −0.29, 0.32; *P* = .93). Overall, this pooled analysis found a small but nonsignificant trend towards the superiority of animal protein (SMD = -0.19; 95% CI: −0.44, 0.05; *P* = .09).

#### Upper Body Strength

Figure S1 ^17^ ^,^ ^32^ ^,^ ^35^ ^,^ ^36^ ^,^ ^38^ ^,^ ^40–42^ ^,^ ^46^ ^,^ ^51^ ^,^ ^52^ ^,^ ^64^ ^,^ ^67^ ^,^ ^69^ ^,^ ^73^ displays a pooled analysis of 14 trials (*n* = 554 participants) that found that effects of plant or animal protein were similar for upper body strength (SMD = -0.12; 95% CI: −0.51, 0.26; *P* = .53). Heterogeneity was high (*I^2^* = 79%). This analysis pooled bench press and hand-grip strength measures; a sensitivity analysis conducted on each of these separate measures of upper body strength did not result in different findings (data not shown). Subgroup analysis by age also did not change the findings (Figure S1).

### Effects on Physical Performance

Figure S2 ^36–38^ ^,^ ^40^ ^,^ ^42^ presents a meta-analysis of 5 trials that measured physical performance, either by Short-Performance Physical Battery (SPPB)^36–38^ or Timed-Up-and-Go (TUG) tests.^36^^,^^40^^,^^42^ One trial presented data for SPPB and TUG; however, only data for SPPB were used in analysis as this was considered to be the optimal measure of physical performance. There was no difference between animal and plant protein interventions on physical performance (SMD = 0.12; 95% CI: −0.21, 0.45; *P* = .47) and heterogeneity was substantial (*I^2^* = 61%).

### Publication Bias

Risk of publication bias was considered low following visual inspection of a funnel plot (Figure S3) and Egger’s test (*P* = .21).

### Narrative Summary of Trials Not Included in Meta-analysis

This section briefly summarizes findings for 11 trials, all conducted in younger adults (<60 years), which could not be included in meta-analysis because outcomes were not appropriate for pooling.^15^^,^^16^^,^^34^^,^^48–50^^,^^54^^,^^56^^,^^66^^,^^68^^,^^70^

Two trials compared pea with milk protein and found no difference in muscle thickness between groups after the intervention period.^15^^,^^16^ A superior effect of soy vs milk protein on muscle mass was observed in young sedentary men,^34^ but these results were not replicated in other studies.^48–50^^,^^56^^,^^66^^,^^70^ A further 2 dietary intervention trials that compared red meat or poultry with lentils and legumes reported no change in percentage of muscle mass.^54^^,^^68^

One trial found that milk protein increased 8-repetition-maximum (8-RM) total strength more than soy protein,^41^ while another trial that compared milk with soy protein found no difference between groups.^53^ Similar increases in strength were found in trials comparing milk protein with pea^15^^,^^16^ and chia seed protein.^69^ A dietary intervention found that a high-protein vegan diet group increased deadlift strength (70% 1-RM) significantly, while the animal protein diet group did not.^24^

No difference was found between soy and milk protein groups for timed rise,^38^^,^^40^^,^^42^ gait speed,^38^ or 6-Minute Walk Test (6MWT).^41^ One trial that compared rice with milk protein reported no difference in gait speed between groups.^36^

## DISCUSSION

To the authors’ knowledge, this is the first systematic review to quantitatively synthesize RCT data relating to the muscle health effects of a range of different plant proteins compared with isonitrogenous animal proteins with analysis of older adults defined as 60 years or older—in line with the WHO and Cochrane-Campbell Global Ageing Partnership definitions of an older adult. The key findings were a small, beneficial pooled effect of animal protein compared with plant protein on muscle mass with no difference between the protein sources for muscle strength or physical performance. In 3 subgroup analyses conducted for the muscle mass outcome, the superior effects of animal protein were apparent in younger (aged <60 years) but not older (≥60 years) adults, in trials that included RT alongside dietary protein and when the plant protein intervention was from a source other than soy. Pooled data from 17 RCTs provided no evidence for a difference in muscle mass between soy protein and milk protein in younger or older groups and in men or in women. In another analysis, animal protein was superior to plant protein for lower body strength (but not upper body strength) in older adults, whereas no difference was seen in younger adults.

Findings from this review involving moderate-to-high-quality trial data indicated that, across longer durations (≥4 weeks), there is little difference in muscle mass with soy protein compared with animal protein, whereas this may not be the case for non-soy plant proteins. A previous meta-analysis of trials 6 or more weeks in duration with RT also concluded that there was no difference between soy and animal protein for muscle mass or strength outcomes.^13^ This may be explained by the high quality of soy plant protein sources with an EAA profile similar to that of milk.^74^^,^^75^ Furthermore, most of the RCTs in the current pooled analysis used soy protein isolates or concentrates. These soy protein sources are known to have the highest DIAAS of all soy products.^76^ It has been noted previously that there appears to be a disconnect between longer-term studies, which found similar effects of soy and milk protein on muscle mass, and acute studies, which suggest a superiority of milk protein on MPS.^74^^,^^75^^,^^77^ It has previously been shown that MPS is a poor predictor of long-term muscle growth.^78^ Long-term studies, such as those included in this review, could therefore be considered a more useful source of data concerning clinically relevant increases in muscle mass. The current review provides the most up-to-date evidence that soy isolate or concentrate is as effective as milk protein for muscle mass maintenance or accrual, even in older adults with poor muscle or functional health.^38^^,^^40^

On the other hand, non-soy plant protein (chia seed, oat, potato, and rice) had a less potent effect on muscle mass compared with milk protein following pooled analysis. The reasons for this are not clear, considering that potato and rice protein isolates have previously demonstrated higher mean EAA contents as a percentage of total protein than soy.^79^ Chia protein has shown intermediate-to-low digestibility^80^ and reduced BCAA content in in vitro models of aged gastrointestinal systems^81^; however, the RCT that tested chia protein was the single trial in this subgroup to find superiority of the plant compared with animal protein intervention for muscle mass.^69^ Further research is warranted considering the paucity of trials that have assessed different non-soy plant proteins, which meant that the independent effects of each protein source could not be separated in meta-analysis, nor could their pooled effects be examined for muscle strength or physical performance. Furthermore, these plant protein sources have good overall nutritional value, particularly chia seeds, which are the richest source of n–3 polyunsaturated fatty acid of any plant food,^82^ and oats, which are high in β-glucan, a digestion-resistant polysaccharide that acts as a substrate for gut microbiota.^83^ Therefore, they remain valuable components of a diet.

Most trials included in this review compared plant with animal protein supplements. However, it is important to examine the effects of plant- vs animal-based dietary patterns, considering the combinations of foods and nutrients within the diet that may act synergistically or antagonistically on physiological mechanisms associated with muscle health. In pooled analysis of 7 trials in which participants changed towards a plant-based diet, including towards a vegan diet^45^ or by replacing most animal protein with plant protein^52^^,^^55^ and specifically with legumes,^35^^,^^39^^,^^58^^,^^62^ there was an adverse effect on muscle mass compared with an isonitrogenous omnivorous diet. This is an important finding, considering that protein is more commonly consumed as food within a diet, rather than as isolated protein supplements. It has been noted previously that plant proteins in their original food matrix may have lower anabolic potential than isolated plant proteins as a result of protein structure.^84^ The secondary structure of plant proteins demonstrates a greater β-sheet conformation, which gives plant proteins hydrophobic properties, facilitating protein aggregation and increasing resistance to proteolysis in the gastrointestinal tract.^85^^,^^86^ Antinutritional factors can also interfere with protein digestion and absorption.^84^ For example, lectins and trypsin and chymotrypsin protease inhibitors are common in pulses^87^ and play an important defensive role in the plant; however, these molecules consequently reduce the bioavailability of protein for human consumption.^88^ There is evidence that preparation—for example, soaking and cooking methods such as boiling, pressure cooking, or microwaving—can influence the concentration of protease inhibitors, thus improving the nutritional profile.^89^ This is further indicated by 1 study reporting that tofu—a minimally processed soy protein source—had the lowest protein quality score of all soy protein sources, while highly processed soy protein concentrate or isolate exhibited the highest protein quality.^76^ This finding suggests that muscle health should be an important consideration in the conversation surrounding sustainable diets and that efforts should be made to optimize muscle anabolism in those moving towards a plant-based diet (eg, by engaging in regular RT).

High-quality protein sources are considered especially important for muscle health in older age to enhance muscle anabolism.^31^ In this review, there was a stronger beneficial effect of animal protein compared with plant protein on muscle mass in trials involving younger adults (<60 years) than in those involving older adults (≥60 years). This is perhaps unexpected, considering that older adults are known to experience age-related anabolic resistance and animal protein sources are considered to have greater anabolic capacity.^84^ One explanation for this finding could be that more trials have been conducted in younger adults (*n* = 21 vs *n* = 9 in older adults); therefore, subgroup analyses in younger adults had a larger sample size, making it possible to detect the small beneficial effect of animal protein on muscle mass. Findings from the current systematic review are in agreement with an earlier meta-analysis that analyzed younger (≤50 years) and older (>50 years) adults separately and found significant lean mass improvements following animal protein, but not plant protein, in younger adults only.^21^ This could also be partly explained by an increased sensitivity of younger muscle to the anabolic stimulus of EAA compared with that in aging muscle,^90^ wherein older adults experienced little change in response to either protein intervention, while younger adults had a stronger response to animal protein.

Evidence suggests that protein supplementation combined with RT is more effective than protein supplementation alone for promoting improvements in muscle mass and strength.^91–93^ Yet, the importance of protein source combined with RT is not well understood. Using the available data, this work demonstrated that the combination of animal protein with RT was more effective for increasing muscle mass than the equivalent plant protein and RT intervention. Furthermore, all 16 trials (100%) that included RT reported a significant improvement in muscle outcomes, while 21% of trials without RT reported a significant change. Strength and physical performance improved with protein and RT only. Therefore, this review supports current evidence that protein in addition to RT is likely to be more effective than protein alone, while also adding to this by showing that animal protein with RT has a small-to-moderate beneficial effect on muscle mass compared with plant protein.

### Strengths, Limitations, and Priorities for Future Research

One key strength of this review is the pooled analysis of 1538 participants across 30 RCTs for muscle mass. This substantial number of trials permitted subgroup analysis based on protein type, which found that non-soy plant proteins may not support muscle mass as well as animal proteins. Another strength is the comprehensive search strategy across 5 key research databases and the implementation of rigorous eligibility criteria. The current review excluded various papers that were presented in another systematic review on this topic—for example, a trial that provided additional EAAs to 1 study arm only,^94^ a short-term trial with 2 weeks’ duration,^95^ and a population undergoing hemodialysis.^96^ This review also utilized the RoB2 tool for quality assessment: the most up-to-date quality-assessment instrument developed and supplied by The Cochrane Collaboration.

Several limitations must also be noted, such as the significant heterogeneity between trials, which is potentially the result of differences in population characteristics, trial duration, exact intervention formulation, protein dose, and timing. While only 1 trial was found to have a high risk of bias, a further 27 out of 43 RCTs (63%) had some quality concerns. There was also a notable lack of trials evaluating the effects of protein sources in sarcopenic or frail older adults. It is plausible that sarcopenic patients may respond differently than healthy individuals, due to differences in factors such as gut microbiota composition^97^ and genetic factors,^98^ which may influence the response to nutritional interventions. There was also inconsistency in the methods used to assess physical performance in the small number of studies that included this as an outcome.

## CONCLUSION

Overall, animal protein had a small, beneficial effect on muscle mass compared with plant protein in younger but not older adults. Subgroup analyses revealed that the stronger muscle-health–promoting effect of animal protein compared with plant protein remained when combined with RT, and when the plant protein intervention was from a source other than soy. Meanwhile, soy plant protein was equivalent to animal protein for maintaining or improving muscle mass in both younger and older adults, and no significant difference was observed between protein sources when no RT was involved, regardless of age group. From the large number of studies focused on soy and milk protein, it is clear that these sources are equally beneficial for muscle mass, yet a dearth of trials assessing other plant protein sources invites future studies to address this research gap. Few trials have tested plant-based dietary interventions alongside RT in sarcopenic or frail patients, which is another considerable gap in the literature.

### Clinical Implication and Future Perspectives

Very few RCTs involved populations with clinically significant low muscle mass or strength; therefore, it is not possible to provide recommendations for this population. In healthy young adults, it appears that animal protein or soy protein in combination with RT is the optimal intervention for increasing muscle mass. It is important to note that any benefits of animal protein over plant protein were slight, as indicated by small effect sizes. At this time, there is little evidence for protein source playing a role in the muscle health of older adults; however, further research in this population is warranted considering the limited number of studies in those aged older than 60 years.

## Supplementary Material

nuae200_Supplementary_Data

## Data Availability

The data used to generate these results were extracted from the trials included in this review and may be accessed directly from the cited papers. If preferable, extracted data will be provided by the authors upon request.
