# Supplementary material for: Effect of Plant Versus Animal Protein on Muscle Mass, Strength, Physical Performance, and Sarcopenia: A Systematic Review and Meta-analysis of Randomized Controlled Trials
Source: Nutr Rev. 2025 Jan 15;83(7):e1581–603. doi: 10.1093/nutrit/nuae200 (PMC12166177; doi:10.1093/nutrit/nuae200)
Supplement: nuae200_Supplementary_Data [file nuae200_supplementary_data.zip › PDF56401354-1160732357.pdf]

Table S1. **Assessment and rates of intervention compliance in 43 included trials.**

Author, year (country)	Assessment of compliance	Intervention compliance
Anderson et al. (2007) [S1] (USA)	Subjects signed an attendance sheet to verify attendance and a staff member inspected the lifestyle diary to record completion. The following information was self-reported by the subjects and recorded on a data sheet at each visit: number of shakes, entrees, and fruits plus vegetables consumed per week. Subjects also estimated their physical activity daily, recorded it in the lifestyle diary, and recorded the weekly total on the data sheet in the clinic. Energy expenditure with physical activity was estimated in kilocalories with the HMR calorie system. All lifestyle calculations were based on active subjects and adjustments were not made for subjects after their withdrawal.	Shake consumption averaged 20.6/wk for casein (98% of goal) and 20.4/wk for soy (97%). One entrée was provided for daily use (7/wk) and entrée consumption averaged 6.8/wk for casein (97%) and 6.9/wk for soy (99%). Subjects were instructed to consume 5 servings of fruits or vegetables daily (≥35/wk) and they reported 54.4 servings per week for casein (155% of goal) and 51.4 servings per week for soy (147% of goal). No differences between groups for compliance.<br><br>Average class attendance was excellent (≥95%) for casein (96%) and soy groups (96%). Lifestyle diary completion averages also were very good (≥90%) to excellent for both groups (96% for casein and 92% for soy).
Babault et al. (2015) [S2] (France)	Compliance assessed by counting products not consumed.	High average and comparable compliance were observed between groups: 93.4%, 90.8% and 90.7% for Whey, Placebo and Pea groups, respectively (*P* = 0.509).
Baer et al. (2011) [S3] (USA)	Compliance was determined by counting the number of packets distributed and recounting those not consumed and by measuring para-amino benzoic acid (PABA) in urine samples collected at random, unannounced times monthly to determine whether PABA was present in the urine. The PABA was added to each treatment packet at a concentration of 0.24 mg/kJ. The half-life of PABA is very short (<18 h); it was decided that ≥3 of 5 urine samples without PABA would be a criterion for noncompliance.	The mean number of supplement packets consumed over the intervention was 2 per day, which was the prescribed amount. However, PABA analysis of urine samples revealed 2 participants with undetectable PABA concentrations in 4 of 5 random samples. Their data were excluded from all the analyses.<br><br>Time of use compliance for physical activity monitors was >72%.
Banaszek et al. (2019) [S4] (USA)	Used supplement packets returned at the end of every week.<br>Resistance training (RT) programme inputted to software at gym after each session.	All dietary recalls and empty supplement bags were returned each week and participants reported full compliance.<br><br>No information on RT compliance.
Basciani et al. (2020) [S5] (Italy)	Daily food diary	Compliance was comparable in all groups. Urine acetoacetic acid, reflecting ketosis, increased significantly from baseline to the end of the VLCKD interventions, and a plateau value was reached after 7 days in all groups.
Barnard et al. (2005) [S6] (USA)	Three-day dietary record using a food scale. Physical activity was assessed with the Bouchard 3-Day Physical Activity Record.	Good compliance suggested by reduced fat and increased carbohydrate/fibre in intervention group. No changes in physical activity.
Beavers et al. (2015) [S7] (USA)	Supplements consumed, body weight and post-intervention fasting serum isoflavone levels used to assess compliance.	Adherence to the dietary protocol was excellent (97.5±3.3% and 92.9±9.3% in the soy and non-soy group, respectively). Biomarker and weight data also corroborated self-reported dietary compliance.
Berger et al. (2014) [S8] (USA)	Adherence was measured in all participants as percent of shakes consumed (number of shake packets/number of days in the study). Adherence was confirmed through assessment of serum isoflavones in a random subsample of participants at baseline (N = 32) and 16 weeks (N = 94).	Percent adherence was not significantly different between groups over the course of the study: adherence was 72% and 77% at 8 weeks, and 69% and 73% at 16 weeks for SOY and CAS groups, respectively.
Brown et al. (2004) [S9] (USA)	The time of the day when the bars were consumed was recorded daily in the subject's fitness log so that compliance could be monitored.	Not reported, however the increases in lean mass in intervention groups but no change in the control group, suggests good compliance.
Candow et al. (2006) [S10] (Canada)	Training programme supervised.	Good compliance.
Christie et al. (2010) [S11] (USA)	Compliance with the supplements was established by measuring serum isoflavone levels at baseline, four weeks, eight weeks, and twelve weeks	Isoflavone levels increased significantly for the treatment group compared to placebo as expected, with increases of 265% in serum genistein and 10,044% in serum daidzein, compared to no changes with placebo, confirming compliance with treatment.
Denysschen et al. (2009) [S12] (USA)	Three-day food records completed at five times across the study period. RT was supervised and subjects were required to participate in > 80% of exercise sessions over the 12-week period. Training logs for each subject were kept by assigned trainers.	Protein intake significantly increased in soy and whey groups compared to a placebo group, indicating good compliance.

Durkalec-Michalski et al. (2022) S13 (Poland)	Participants recorded daily dietary intake on MyFitnessPal mobile app. Participants also had contact with a sports dietitian three times per week. Exercise was supervised by a personal trainer.	Potential participants who did not declare diet adherence and compliance with the study protocol throughout the duration of the study were excluded from the recruitment.
Evans et al. (2007) S14 (USA)	No information.	Stated in results section that subjective data indicated that adherence to the protein supplement was approximately 90%.
Gonzales-Salazar et al. (2021) S15 (Mexico)	Compliance with the diet was monitored through a 24-h reminder carried out in person each week and by twice weekly telephone calls with nutritionists. Participants recorded what they consumed in food logs 3 days a week.	The percentage of compliance to dietary treatment was 96.5% in the Animal NP, 97.1% Vegetable NP, 94% Animal HP and 95.7% in the Vegetable HP group (P = 0.373).
Hartman et al. (2007) S16 (Canada)	RT was supervised to ensure compliance. Protein drinks were consumed under supervision, before and after exercise.	Compliance with the prescribed training protocol was 86 ± 2% ($\bar{x}$ ± SEM) overall with all subjects achieving ≥75% compliance and 13 of 54 subjects had 100% compliance (ie, attendance at and completion of every training session).
Hashimoto et al. (2015) S17 (Japan)	Participants were asked to record details of their diet on "brief-type self-administered diet history questionnaires" (BDHQ).	No information.
Hassanzadeh-Rostami et al. (2019) S18 (Iran)	Participants were visited twice and had weekly calls to monitor compliance. They also completed three 24-hour food records.	Good adherence based on dietary intake reports.
Haub et al. (2002) S19 (USA)	The research team asked the participants about their adherence throughout the intervention and provided advice if needed. RT sessions were supervised by an exercise specialist.	Subjects in the BC group consumed 57 ± 10% of their total protein from the supplemental beef and subjects in the LOV group consumed 53 ± 7% of their total protein from the supplemental texturized vegetable protein during the 12-wk period of resistive training, suggesting good compliance.
Hill et al. (2015) S20 (USA)	Compliance monitored through daily questionnaires. Participants were classified as noncompliant on any day that they consumed a non-study food or beverage or did not consume a study food or beverage.	Compliance ranged from 70–90% of study days.
Jadczak et al. (2021) S21 (Australia)	Compliance diaries for exercise and protein supplements were completely weekly for the first 2 weeks, and then every 2 weeks. If a problem with compliance was identified, the study team interviewed the participant to assess any difficulties and identify strategies to facilitate compliance.	The rate of compliance was 96.2% for the exercise and nutrition protocol. Compliance to the 2x/day protein sachets was 90.3%. Compliance did not differ between groups. In terms of exercise, compliance to the centre-based exercise class was low with 50.9% attending at least 70% of the exercise classes during the 6 months. In contrast, compliance to the home-based exercise sessions (148.3%) as well as walking (179.3%) was high.
Joy et al. (2013) S22 (USA)	Protein supplement ingestion was supervised following RT. RT sessions were 'closely monitored' to ensure compliance with effort and intensity.	Rates of compliance reported.
Kalman et al. (2007) S23 (USA)	Compliance to supplements was monitored through return of empty packets to the laboratory on a weekly basis. One of three RT sessions per week was supervised.	Only the soy group significantly increased protein intake. Analysis was completed on those who had ≥50% compliance to the supplements and 100% compliance to the RT programme.
Kjolbaek et al. (2017) S24 (Denmark)	Compliance was tested by 24-hour urinary nitrogen excretion and participants outside of the set cut-off were excluded from analysis.	Significantly higher protein intake in each intervention group compared to control group, suggesting good compliance. Poorly compliant participants were excluded from analysis.
Kok et al. (2006) S25 (Netherlands)	Plasma genistein levels and dietary records.	Compliance reported to be good: 90% of the participants took the supplement for approximately 80% of the intervention period.
Li et al. (2016) S26 (USA)	Noncompliance to the diet was reported using a daily checklist. Compliance was also assessed using body weight and blood urea nitrogen. Noncompliant participants were excluded from analysis.	Participants were compliant to the intervention.
Li et al. (2021) S27 (China)	Compliance was measured by counting empty supplement packets.	The compliance rate was 99.0% and 91.5% for whey and soy protein, respectively.
Liu et al. (2010) S28 (Hong Kong)	Compliance was measured by counting empty supplement packets.	Adherence was good and did not differ among the three groups. More than 90% of the subjects used 97% of the supplements.
Liu et al. (2013) S29 (Hong Kong)	Compliance was measured by counting empty supplement packets, 24-hour urinary excretion of isoflavones and food diaries.	Adherence was good (suggested by urinary isoflavones) and did not differ among the 3 groups. Compliance was 93.3% in the whole soy group and 92.9% in the animal protein group.

Lukaszuk et al. (2007) [S30] (USA)	Compliance was assessed using 3-day food records and exercise logs.	No difference in the increased protein intakes of each arm.
Markova et al. (2016) [S31] (Germany)	All dietary intake documented and weighed.	Adherence to dietary plans was good with similar compliances for both diets (AP: 29.5 EN% protein, 40.4 EN% carbohydrates, 30.1 EN% fat; PP: 29.9 EN% protein, 39.2 EN% carbohydrates, 30.9 EN% fat).
McBreairty et al. (2020) [S32] (Canada)	Dietary intake was recorded in a daily logbook.	Women in the pulse group followed the guidelines 5.48 ± 0.36 days per week. The TLC group followed the guidelines a mean of 5.30 ± 0.26 days/week. The compliance to dietary interventions was comparable between groups.
Moeller et al. (2003) [S33] (USA)	Adherence measured through urinary isoflavone excretion and self-reported consumption of supplements.	Urinary isoflavone excretion and self-reported consumption of muffins and powder indicated excellent adherence to dietary treatment.
Moon et al. (2020) [S34] (USA)	Participants were removed from analysis if their compliance to protein or RT regimens fell below <90%. Participants completed a training log each week. Dietary compliance was tracked using MyFitnessPal mobile app. Supplement compliance was monitored by returning empty sachets/with a photograph of empty sachets.	Compliance to recording their workout information and submitting pictures was determined to be 99.8% for the whey protein group and 99.4% for the rice protein group. Compliance to the supplementation protocol was calculated to be 99.4% for the whey protein group and 99.6% for the rice protein group.
Pettersson et al. (2021) [S35] (Sweden)	Compliance to drink ingestion was confirmed by continuous glucose monitoring. Exercise compliance was monitored via supervised sessions.	Compliance was 99.8% of the planned training, and the mean training load did not differ between groups.
Roschel et al. (2021) [S36] (Brazil)	Compliance was monitored by weighing the remaining supplement in containers at the end of the study. RT was supervised.	Unclear, however subgroup analysis was conducted with participants who had >70% compliance and results were not different to the main analysis.
Sites et al. (2007) [S37] (USA)	Not reported.	Not reported.
Thomson et al. (2016) [S38] (Australia)	Daily food checklists and urinary urea and creatinine were used to monitor compliance. Exercise was monitored using training logs.	Average protein intake was at least 1.2 grams per kilogram body weight per day, in the plant and animal study arms.
Volek et al. (2013) [S39] (USA)	Supplements were consumed in front of study personnel on training days. Participants also kept a log with the date and time that supplements were taken. Empty supplement packets were counted every two weeks. Supplements were laced with para-aminobenzoic acid (PABA) and unannounced urine samples were taken to monitor compliance.	Based on PABA levels, overall compliance was 82%. Only those with a compliance of 90% were analysed.
Vupadhyayula et al. (2009) [S40] (USA)	Consumed protein supplement packets were counted at the clinic.	Compliance was high at 95% (95.4% in the soy protein, 95.5% in the soy isoflavone, and 96.3% in the milk protein group) over 2 years in those who completed the intervention.
Wilson et al. (2022) [S41] (USA)	Not reported.	Not reported.
Zbinden-Foncea et al. (2023) [S42] (Chile)	Compliance was assessed using a survey at the end of the study. RT was supervised.	According to the survey, all participants consumed their supplements after their training sessions.
Zemel et al. (2010) [S43] (USA)	Participants maintained diet and physical activity logs throughout the study. Two of three daily protein supplements were supervised.	Not reported.

Figure S1. Meta-analysis of change in upper body strength in response to plant versus animal protein interventions, overall and in younger (<60 years) and older (≥60 years) adults.

	Plant			Animal				Std. Mean Difference
Study or Subgroup	Mean	SD	Total	Mean	SD	Total	Weight	IV, Random, 95% CI
**11.7.1 Younger adults**								
Volek et al. (2013)[S39]	15.9	1.4	22	20.1	2.3	19	6.4%	−2.20 [−3.00, −1.41]
Basciani et al. (2020)[S5]	0.88	1.99	16	1.98	2.24	16	6.8%	−0.51 [−1.21, 0.20]
Zbinden−Foncea et al. (2023)[S42]	21	8	6	22	10	6	5.0%	−0.10 [−1.23, 1.03]
Joy et al. (2013)[S22]	9.6	5.96	12	9	12.59	12	6.4%	0.06 [−0.74, 0.86]
DeNysschen et al. (2009)[S12]	18.2	8.16	10	17.4	8.3	9	5.9%	0.09 [−0.81, 0.99]
Moon et al. (2020)[S34]	4.1	5.45	12	2.6	4.43	12	6.3%	0.29 [−0.51, 1.10]
Gonzales−Salazar et al. (2021) (i)[S15]*	1.2	2.79	18	−0.9	2.12	18	6.9%	0.83 [0.14, 1.51]
Gonzales−Salazar et al. (2021) (ii)[S15]*	0.8	1.89	20	−1.7	3.09	19	6.9%	0.96 [0.30, 1.63]
**Subtotal (95% CI)**			**116**			**111**	**50.6%**	**−0.06 [−0.78, 0.65]**
Heterogeneity: $Tau^2 = 0.89$; $Chi^2 = 45.87$, df = 7 (P < 0.00001); $I^2 = 85\%$								
Test for overall effect: Z = 0.17 (P = 0.86)								
**11.7.2 Older adults**								
Haub et al. (2002)[S19]	62	16.99	11	104	39	10	5.6%	−1.37 [−2.34, −0.39]
Roschel et al. (2021)[S36]	2	3.14	22	5	2.87	22	7.1%	−0.98 [−1.61, −0.35]
Thomson et al. (2016)[S38]	17.4	11.1	26	17.4	6.7	34	7.6%	0.00 [−0.51, 0.51]
Jadczak et al. (2021)[S21]	2.5	2.93	30	2.3	3.85	21	7.4%	0.06 [−0.50, 0.62]
Beavers et al. (2015)[S7]	0.2	9.3	12	−0.9	9	12	6.4%	0.12 [−0.68, 0.92]
Vupadhyayula et al. (2009)[S40]	−0.67	2.19	30	−1.04	3.14	35	7.7%	0.13 [−0.35, 0.62]
Li et al. (2021)[S27]	0.6	1.99	31	−0.28	2.31	31	7.6%	0.40 [−0.10, 0.91]
**Subtotal (95% CI)**			**162**			**165**	**49.4%**	**−0.16 [−0.57, 0.25]**
Heterogeneity: $Tau^2 = 0.21$; $Chi^2 = 19.42$, df = 6 (P = 0.004); $I^2 = 69\%$								
Test for overall effect: Z = 0.78 (P = 0.44)								
**Total (95% CI)**			**278**			**276**	**100.0%**	**−0.12 [−0.51, 0.26]**
Heterogeneity: $Tau^2 = 0.44$; $Chi^2 = 65.42$, df = 14 (P < 0.00001); $I^2 = 79\%$								
Test for overall effect: Z = 0.62 (P = 0.53)								
Test for subgroup differences: $Chi^2 = 0.06$, df = 1 (P = 0.81), $I^2 = 0\%$								

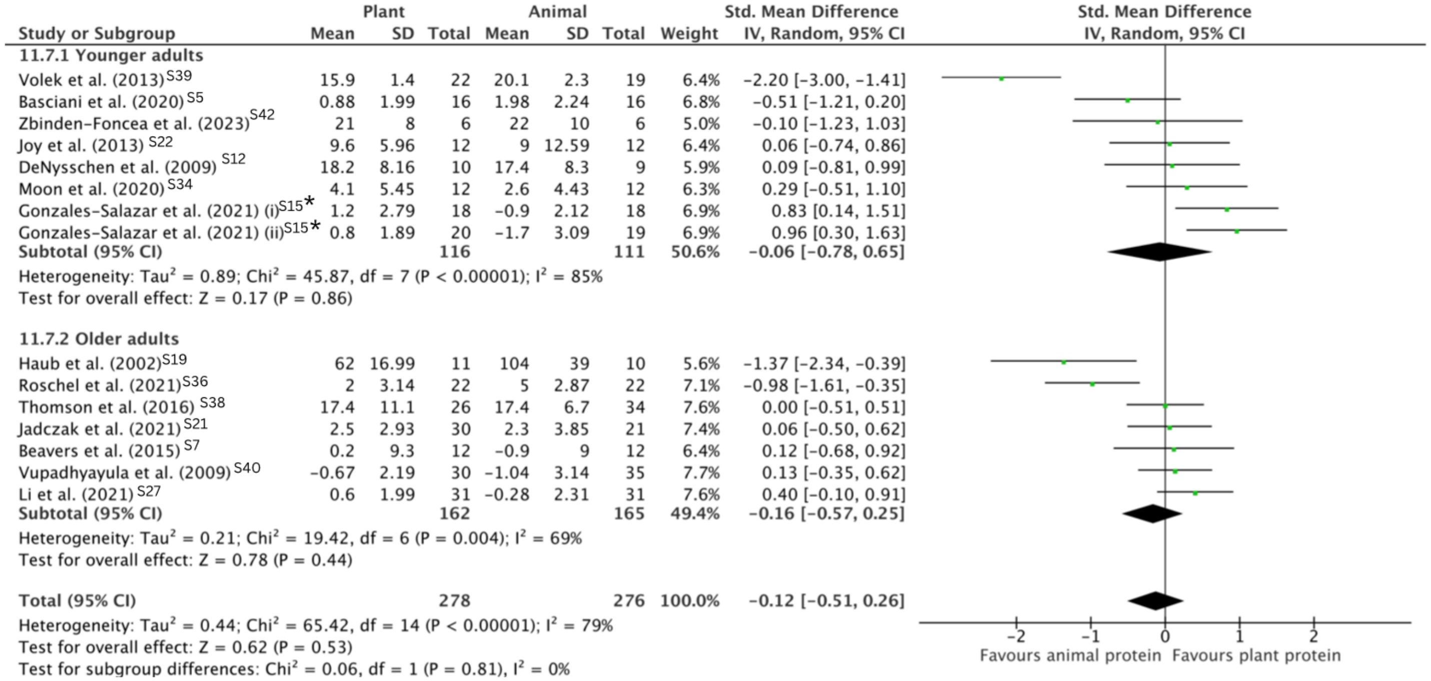

*Gonzales−Salazar et al. (2021) (i) denotes the groups who received a normal protein diet (19% of daily energy), and (ii) denotes those who received a high protein diet (29% of daily energy).

Figure S2. Meta-analysis of change in physical performance in response to plant versus animal protein interventions.

	Plant			Animal				Std. Mean Difference
Study or Subgroup	Mean	SD	Total	Mean	SD	Total	Weight	IV, Random, 95% CI
Vupadhyayula et al. (2009)[S40]	−0.42	1.25	39	−0.2	0.85	37	20.7%	−0.20 [−0.65, 0.25]
Kok et al. (2005)[S25]	0.07	0.87	100	0.18	0.87	102	27.4%	−0.13 [−0.40, 0.15]
Jadczak et al. (2021)[S21]	0.4	0.87	30	0.4	0.87	23	17.5%	0.00 [−0.54, 0.54]
Roschel et al. (2021)[S36]	0.2	1.25	22	−0.3	0.85	22	15.9%	0.46 [−0.14, 1.06]
Li et al. (2021)[S27]	0.47	0.87	31	−0.12	0.87	31	18.5%	0.67 [0.16, 1.18]
**Total (95% CI)**			**222**			**215**	**100.0%**	**0.12 [−0.21, 0.45]**

Heterogeneity: $Tau^2 = 0.08$; $Chi^2 = 10.20$, df = 4 (P = 0.04); $I^2 = 61\%$
Test for overall effect: Z = 0.72 (P = 0.47)

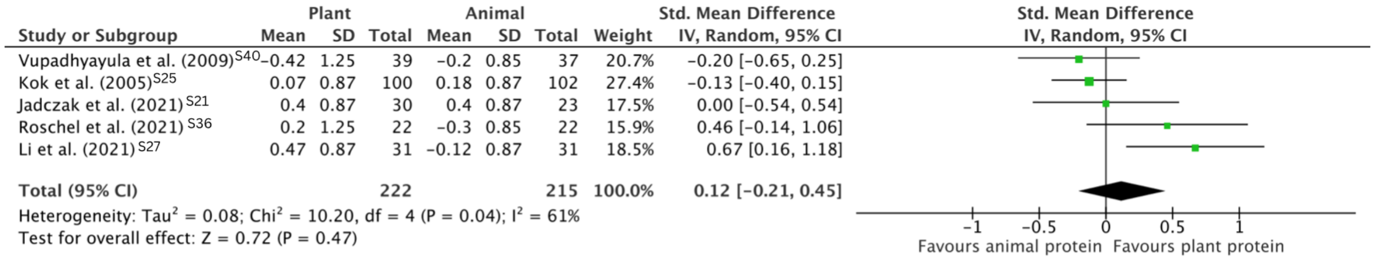

Figure S3. Funnel plot to examine risk of publication bias for muscle mass in 30 trials included in meta-analysis *

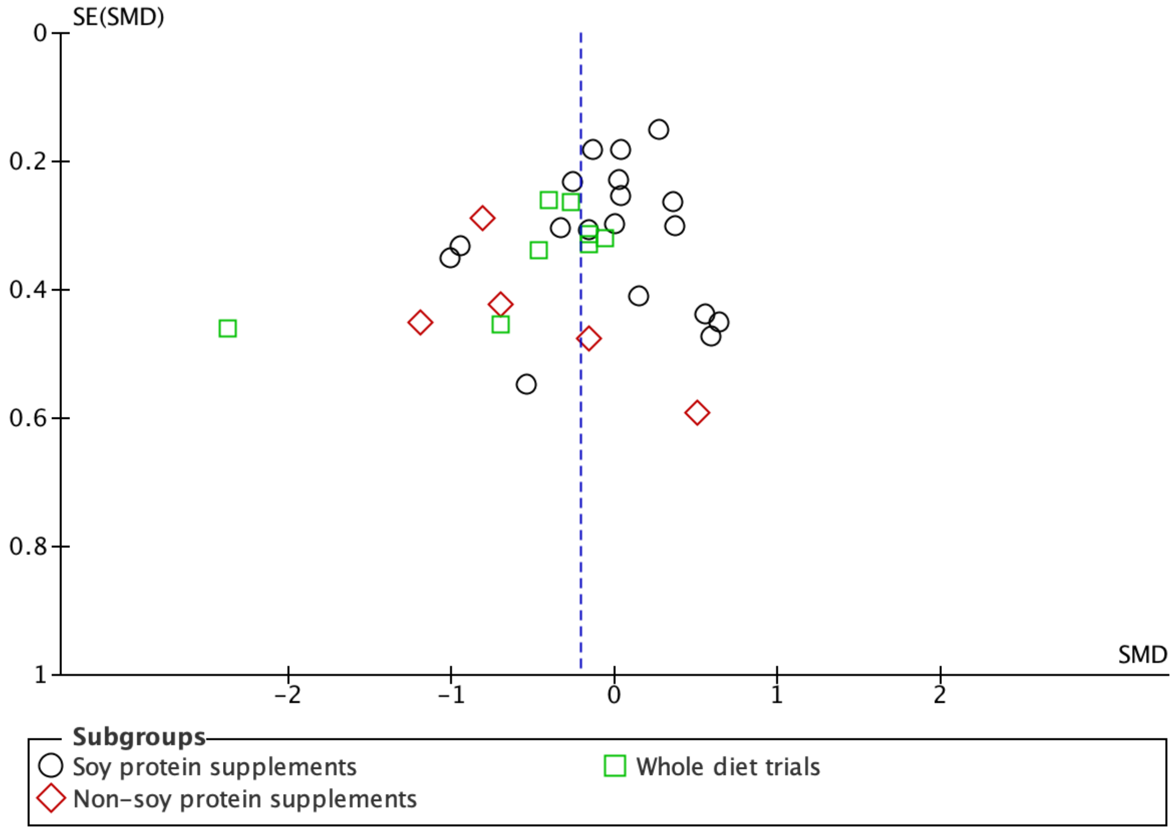

SE = Standard error; SMD = Standardised mean difference.

* Negative values indicate favourability of animal protein.

Supplementary material references

S1. Anderson JW, Fuller J, Patterson K, Blair R, Tabor A. Soy compared to casein meal replacement shakes with energy-restricted diets for obese women: randomized controlled trial. *Metabolism*. 2007;56(2):280-8. doi:10.1016/j.metabol.2006.10.013.

S2. Babault N, Païzis C, Deley G, et al. Pea proteins oral supplementation promotes muscle thickness gains during resistance training: a double-blind, randomized, Placebo-controlled clinical trial vs. Whey protein. *J Int Soc Sports Nutr*. 2015;12(1):3. doi:10.1186/s12970-014-0064-5.

S3. Baer DJ, Stote KS, Paul DR, Harris GK, Rumpler WV, Clevidence BA. Whey protein but not soy protein supplementation alters body weight and composition in free-living overweight and obese adults. *J Nutr*. 2011;141(8):1489-94. doi:10.3945/jn.111.139840.

S4. Banaszek A, Townsend JR, Bender D, Vantrease WC, Marshall AC, Johnson KD. The Effects of Whey vs. Pea Protein on Physical Adaptations Following 8-Weeks of High-Intensity Functional Training (HIFT): A Pilot Study. *Sports (Basel)*. 2019;7(1). doi:10.3390/sports7010012.

S5. Basciani S, Camajani E, Contini S, et al. Very-Low-Calorie Ketogenic Diets With Whey, Vegetable, or Animal Protein in Patients With Obesity: A Randomized Pilot Study. *J Clin Endocrinol Metab*. 2020;105(9)doi:10.1210/clinem/dgaa336.

S6. Barnard ND, Scialli AR, Turner-McGrievy G, Lanou AJ, Glass J. The effects of a low-fat, plant-based dietary intervention on body weight, metabolism, and insulin sensitivity. *Am J Med*. 2005;118(9):991-7. doi:10.1016/j.amjmed.2005.03.039.

S7. Beavers KM, Gordon MM, Easter L, et al. Effect of protein source during weight loss on body composition, cardiometabolic risk and physical performance in abdominally obese, older adults: a pilot feeding study. *J Nutr Health Aging*. Jan 2015;19(1):87-95. doi:10.1007/s12603-015-0438-7.

S8. Berger PK, Principe JL, Laing EM, et al. Weight gain in college females is not prevented by isoflavone-rich soy protein: a randomized controlled trial. *Nutr Res*. 2014;34(1):66-73. doi:10.1016/j.nutres.2013.09.005.

S9. Brown EC, DiSilvestro RA, Babaknia A, Devor ST. Soy versus whey protein bars: Effects on exercise training impact on lean body mass and antioxidant status. *Nutrition Journal*. 2004;3(1):22. doi:10.1186/1475-2891-3-22.

S10. Candow DG, Burke NC, Smith-Palmer T, Burke DG. Effect of whey and soy protein supplementation combined with resistance training in young adults. *Int J Sport Nutr Exerc Metab*. Jun 2006;16(3):233-44. doi:10.1123/ijsnem.16.3.233.

S11. Christie DR, Grant J, Darnell BE, Chapman VR, Gastaldelli A, Sites CK. Metabolic effects of soy supplementation in postmenopausal Caucasian and African American women: a randomized, placebo-controlled trial. *Am J Obstet Gynecol*. Aug 2010;203(2):153.e1-9. doi:10.1016/j.ajog.2010.02.058.

S12. Denysschen CA, Burton HW, Horvath PJ, Leddy JJ, Browne RW. Resistance training with soy vs whey protein supplements in hyperlipidemic males. *J Int Soc Sports Nutr*. 2009;6:8. doi:10.1186/1550-2783-6-8.

S13. Durkalec-Michalski K, Domagalski A, Główka N, Kamińska J, Szymczak D, Podgórski T. Effect of a Four-Week Vegan Diet on Performance, Training Efficiency and Blood Biochemical Indices in CrossFit-Trained Participants. *Nutrients*. 2022;14(4):894.

S14. Evans EM, Racette SB, Van Pelt RE, Peterson LR, Villareal DT. Effects of soy protein isolate and moderate exercise on bone turnover and bone mineral density in postmenopausal women. *Menopause*. 2007;14(3 Pt 1):481-8. doi:10.1097/01.gme.0000243570.78570.f7.

S15. González-Salazar LE, Pichardo-Ontiveros E, Palacios-González B, et al. Effect of the intake of dietary protein on insulin resistance in subjects with obesity: a randomized controlled clinical trial. *Eur J Nutr*. 2021;60(5):2435-2447. doi:10.1007/s00394-020-02428-5.

S16. Hartman JW, Tang JE, Wilkinson SB, et al. Consumption of fat-free fluid milk after resistance exercise promotes greater lean mass accretion than does consumption of soy or carbohydrate in young, novice, male weightlifters. *Am J Clin Nutr*. 2007;86(2):373-81. doi:10.1093/ajcn/86.2.373.

S17. Hashimoto R, Sakai A, Murayama M, et al. Effects of dietary soy protein on skeletal muscle volume and strength in humans with various physical activities. *J Med Invest*. 2015;62(3-4):177-83. doi:10.2152/jmi.62.177.

S18. Hassanzadeh-Rostami Z, Hemmatdar Z, Pishdad GR, Faghih S. Moderate Consumption of Red Meat, Compared to Soy or Non-Soy Legume, Has No Adverse Effect on Cardio-Metabolic Factors in Patients with Type 2 Diabetes. *Exp Clin Endocrinol Diabetes*. 2019;129(06):429-437. doi:10.1055/a-0929-6287.

S19. Haub MD, Wells AM, Tarnopolsky MA, Campbell WW. Effect of protein source on resistive-training-induced changes in body composition and muscle size in older men. *Am J Clin Nutr*. 2002;76(3):511-7. doi:10.1093/ajcn/76.3.511.

S20. Hill AM, Harris Jackson KA, Roussell MA, West SG, Kris-Etherton PM. Type and amount of dietary protein in the treatment of metabolic syndrome: a randomized controlled trial. *Am J Clin Nutr*. 2015;102(4):757-70. doi:10.3945/ajcn.114.104026.

S21. Jadczak AD, Visvanathan R, Barnard R, Luscombe-Marsh N. A Randomized Controlled Pilot Exercise and Protein Effectiveness Supplementation Study (EXPRESS) on Reducing Frailty Risk in Community-Dwelling Older People. *Journal of Nutrition in Gerontology and Geriatrics*. 2021/01/02 2021;40(1):26-45. doi:10.1080/21551197.2021.1886222.

S22. Joy JM, Lowery RP, Wilson JM, et al. The effects of 8 weeks of whey or rice protein supplementation on body composition and exercise performance. *Nutr J*. 2013;12:86. doi:10.1186/1475-2891-12-86.

S23. Kalman D, Feldman S, Martinez M, Krieger DR, Tallon MJ. Effect of protein source and resistance training on body composition and sex hormones. *J Int Soc Sports Nutr*. 2007;4:4. doi:10.1186/1550-2783-4-4.

S24. Kjølbæk L, Sørensen LB, Søndertoft NB, et al. Protein supplements after weight loss do not improve weight maintenance compared with recommended dietary protein intake despite beneficial effects on appetite sensation and energy expenditure: a randomized, controlled, double-blinded trial. *Am J Clin Nutr*. 2017;106(2):684-697. doi:10.3945/ajcn.115.129528.

S25. Kok L, Kreijkamp-Kaspers S, Grobbee DE, Lampe JW, van der Schouw YT. Soy isoflavones, body composition, and physical performance. *Maturitas*. 2005;52(2):102-10. doi:10.1016/j.maturitas.2005.01.003.

S26. Li J, Armstrong CL, Campbell WW. Effects of Dietary Protein Source and Quantity during Weight Loss on Appetite, Energy Expenditure, and Cardio-Metabolic Responses. *Nutrients*. 2016;8(2):63. doi:10.3390/nu8020063.

S27. Li C, Meng H, Wu S, et al. Daily Supplementation With Whey, Soy, or Whey-Soy Blended Protein for 6 Months Maintained Lean Muscle Mass and Physical Performance in Older Adults With Low Lean Mass. *Journal of the Academy of Nutrition and Dietetics*. 2021;121(6):1035-1048.e6. doi:https://doi.org/10.1016/j.jand.2021.01.006.

S28. Liu ZM, Ho SC, Chen YM, Ho YP. A mild favorable effect of soy protein with isoflavones on body composition-a 6-month double-blind randomized placebo-controlled trial among Chinese postmenopausal women. *Int J Obes (Lond)*. 2010;34(2):309-18. doi:10.1038/ijo.2009.236.

S29. Liu ZM, Ho SC, Chen YM, Woo J. A six-month randomized controlled trial of whole soy and isoflavones daidzein on body composition in equol-producing postmenopausal women with prehypertension. *J Obes*. 2013;2013:359763. doi:10.1155/2013/359763.

S30. Lukaszuk JM, Luebbers P, Gordon BA. Preliminary study: soy milk as effective as skim milk in promoting weight loss. *J Am Diet Assoc*. 2007;107(10):1811-4. doi:10.1016/j.jada.2007.07.008.

S31. Markova M, Pivovarova O, Hornemann S, et al. Isocaloric Diets High in Animal or Plant Protein Reduce Liver Fat and Inflammation in Individuals With Type 2 Diabetes. *Gastroenterology*. 2017;152(3):571-585.e8. doi:10.1053/j.gastro.2016.10.007.

S32. McBreairty LE, Kazemi M, Chilibeck PD, Gordon JJ, Chizen DR, Zello GA. Effect of a pulse-based diet and aerobic exercise on bone measures and body composition in women with polycystic ovary syndrome: A randomized controlled trial. *Bone Reports*. 2020;12:100248. doi:https://doi.org/10.1016/j.bonr.2020.100248.

S33. Moeller LE, Peterson CT, Hanson KB, et al. Isoflavone-rich soy protein prevents loss of hip lean mass but does not prevent the shift in regional fat distribution in perimenopausal women. *Menopause*. 2003;10(4):322-31. doi:10.1097/01.Gme.0000054763.94658.Fd.

S34. Moon JM, Ratliff KM, Blumkaitis JC, et al. Effects of daily 24-gram doses of rice or whey protein on resistance training adaptations in trained males. *Journal of the International Society of Sports Nutrition*. 2020;17(1):60. doi:10.1186/s12970-020-00394-1.

S35. Pettersson S, Edin F, Hjelte C, et al. Six Weeks of Aerobic Exercise in Untrained Men With Overweight/Obesity Improved Training Adaptations, Performance and Body Composition Independent of Oat/Potato or Milk Based Protein-Carbohydrate Drink Supplementation. Original Research. *Frontiers in Nutrition*. 2021;8. doi:10.3389/fnut.2021.617344.

S36. Roschel H, Hayashi AP, Fernandes AL, et al. Supplement-based nutritional strategies to tackle frailty: A multifactorial, double-blind, randomized placebo-controlled trial. *Clinical Nutrition*. 2021;40(8):4849-4858. doi:https://doi.org/10.1016/j.clnu.2021.06.024.

S37. Sites CK, Cooper BC, Toth MJ, Gastaldelli A, Arabshahi A, Barnes S. Effect of a daily supplement of soy protein on body composition and insulin secretion in postmenopausal women. *Fertil Steril*. 2007;88(6):1609-17. doi:10.1016/j.fertnstert.2007.01.061.

S38. Thomson RL, Brinkworth GD, Noakes M, Buckley JD. Muscle strength gains during resistance exercise training are attenuated with soy compared with dairy or usual protein intake in older adults: A randomized controlled trial. *Clin Nutr*. 2016;35(1):27-33. doi:10.1016/j.clnu.2015.01.018.

S39. Volek JS, Volk BM, Gómez AL, et al. Whey protein supplementation during resistance training augments lean body mass. *J Am Coll Nutr*. 2013;32(2):122-35. doi:10.1080/07315724.2013.793580.

S40. Vupadhyayula PM, Gallagher JC, Templin T, Logsdon SM, Smith LM. Effects of soy protein isolate on bone mineral density and physical performance indices in postmenopausal women-a 2-year randomized, double-blind, placebo-controlled trial. *Menopause*. 2009;16(2):320-8. doi:10.1097/gme.0b013e3181844893.

S41. Wilson SMG, Peterson EJ, Gaston ME, Kuo WY, Miles MP. Eight weeks of lentil consumption attenuates insulin resistance progression without increased gastrointestinal symptom severity: A randomized clinical trial. *Nutr Res*. 2022;106:12-23. doi:10.1016/j.nutres.2022.08.002.

S42. Zbinden-Foncea H, Ramos-Navarro C, Hevia-Larraín V, et al. Neither Chia Flour nor Whey Protein Supplementation Further Improves Body Composition or Strength Gains after a Resistance Training Program in Young Subjects with a Habitual High Daily Protein Intake. *Nutrients*. 2023;15(6). doi:10.3390/nu15061365.

S43. Zemel MB, Sun X, Sobhani T, Wilson B. Effects of dairy compared with soy on oxidative and inflammatory stress in overweight and obese subjects. *Am J Clin Nutr*. 2010;91(1):16-22. doi:10.3945/ajcn.2009.28468.
