# Supplementary figures and images for: Effect of Plant Versus Animal Protein on Muscle Mass, Strength, Physical Performance, and Sarcopenia: A Systematic Review and Meta-analysis of Randomized Controlled Trials
Source: Nutr Rev. 2025 Jan 15;83(7):e1581–603. doi: 10.1093/nutrit/nuae200 (PMC12166177; doi:10.1093/nutrit/nuae200)

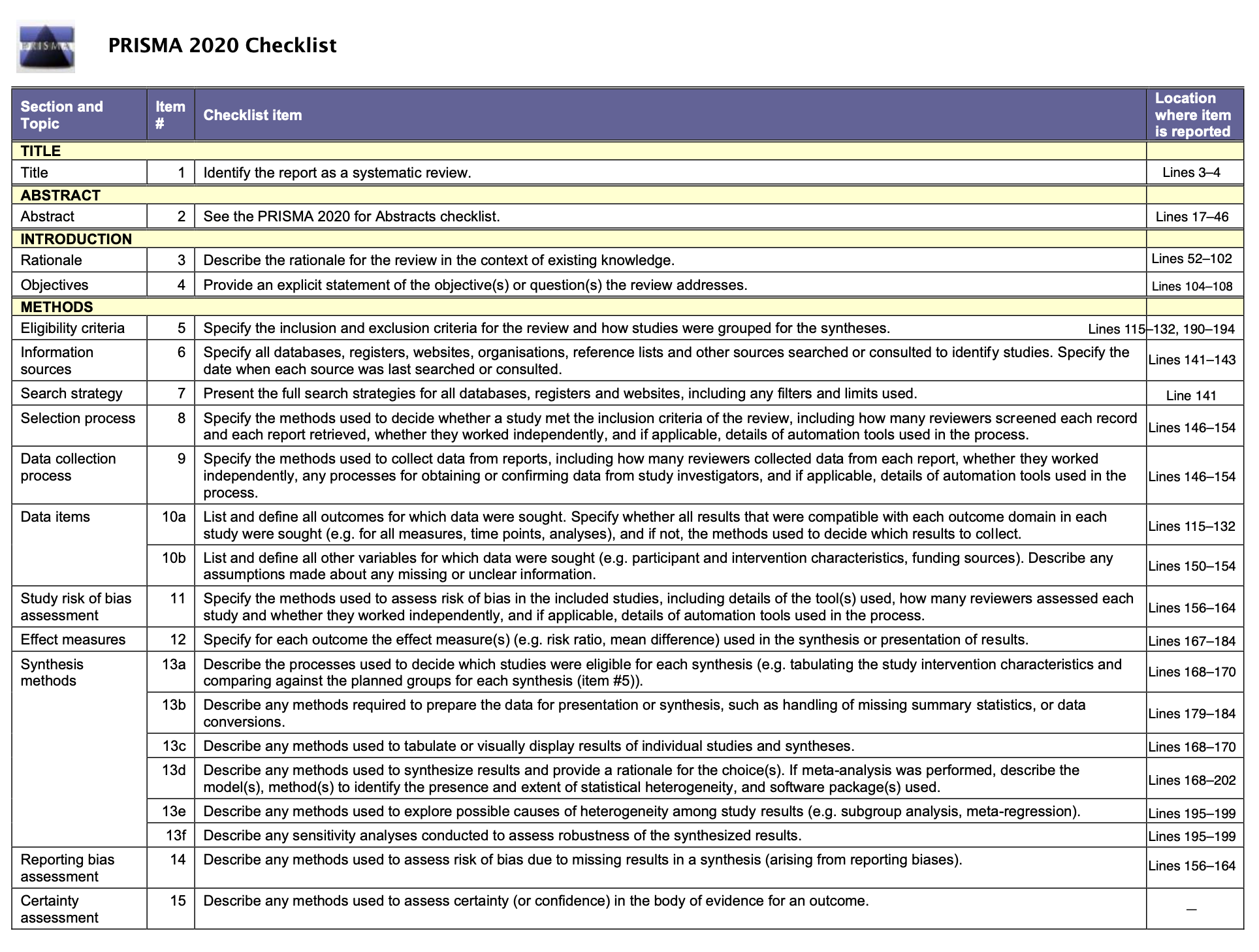


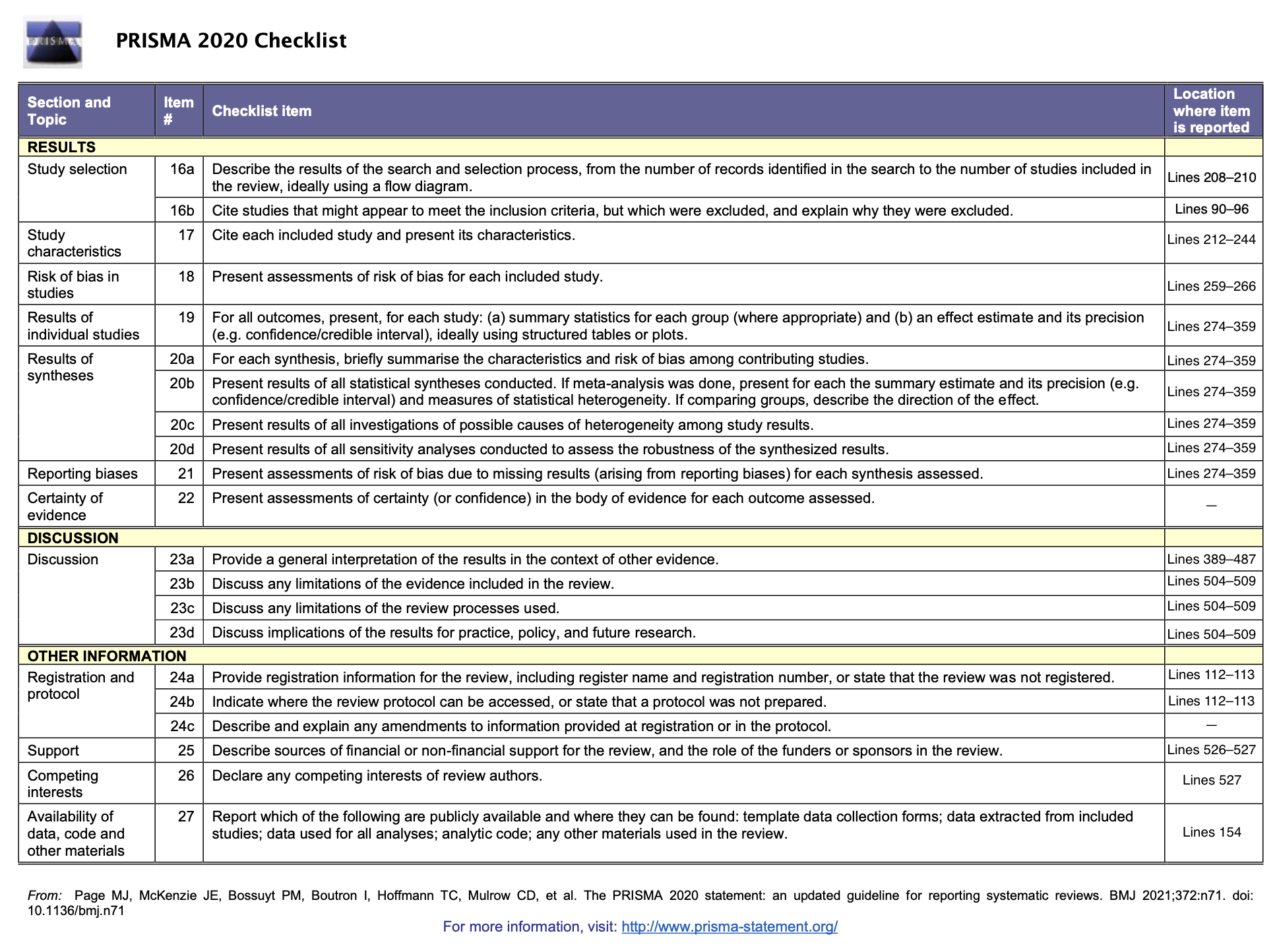


Lines 501-504

Supplement: nuae200_Supplementary_Data [file nuae200_supplementary_data.zip › Supplementary material_PRISMA_checklist.docx]
